# Fast food consumption among young adolescents aged 12–15 years in 54 low- and middle-income countries

**DOI:** 10.1080/16549716.2020.1795438

**Published:** 2020-08-07

**Authors:** Lian Li, Ning Sun, Lina Zhang, Guodong Xu, Jingjing Liu, Jingcen Hu, Zhiying Zhang, Jianjun Lou, Hongxia Deng, Zhisen Shen, Liyuan Han

**Affiliations:** a Department of Epidemiology, Zhejiang Provincial Key Laboratory of Pathophysiology, School of Medicine, Ningbo University, Ningbo, China; b School of Nursing, Ningbo College of Health Sciences, Ningbo, Zhejiang, PR China; c Department of Social Medicine, School of Public Health, Harbin Medical University, Harbin, China; d Teaching and Research Department, Affiliated Hospital of Weifang Medical University, Weifang, China; e Nutrition Department, Yuyao People’s Hospital, Yuyao, Zhejiang, PR China; f Department of Otorhinolaryngology Head and Neck Surgery, Ningbo Medical Center Lihuili Hospital, Ningbo, Zhejiang, PR China; g Hwa Mei Hospital, University of Chinese Academy of Sciences, Ningbo, Zhejiang, PR China; h Department of Global Health, Ningbo Institute of Life and Health Industry, University of Chinese Academy of Sciences, Ningbo, Zhejiang, PR China

**Keywords:** Fast food, adolescents, low- and middle-income countries, GSHS

## Abstract

**Background:**

Most countries worldwide, especially low- and middle-income countries (LMICs), are facing an increasing prevalence of fast-food consumption and multiple burdens of malnutrition among young adolescents.

**Objective:**

To compare the prevalence of fast-food consumption among young adolescents in LMICs.

**Methods:**

We used data from the most recent Global School-Based Student Health Survey (2009–2015), which had been collected using a standardized questionnaire. The weighted prevalence and 95% confidential intervals of fast-food consumption were calculated overall and in subgroups stratified by age, sex, and nutritional status. The pooled overall and regional estimates were obtained using a random-effects model. Heterogeneity was assessed using the I2 statistic. The associated risk factors for fast-food consumption were explored using logistic regression analysis.

**Results:**

Our study comprised 153,496 young adolescents (46.90% boys) from 54 LMICs. Overall, approximately 55.2% (51.3–59.1%) of the adolescents consumed fast food at least 1 day per week, and 10.3% (8.3–12.4%) did so 4–7 days per week. The prevalence of fast-food consumption 4–7 days per week was lowest in the Americas (8.3%; 6.7–9.9%) and highest in Southeast Asia (17.7%; 2.3–33.2%). At a country level, the prevalence was lowest in Pakistan (1.5%; 1.0–2.0%) and highest in Thailand (43.3%; 40.4–46.1%). Furthermore, in subgroups stratified by nutritional status, the prevalence was lowest in the obesity group (6.6%; 4.5–8.7%). Factors such as age, sex, BMI, food insecurity, fruit consumption, vegetable consumption, soft-drink consumption, smoking, physical activity level, and sedentary behavior level were found to be correlated with fast-food consumption.

**Conclusions:**

The identified high prevalence of fast-food consumption among young adolescents in LMICs indicates the urgent need to prioritize the implementation of healthy-diet promotion programs to improve adolescent health in these countries.

## Background

Globally, young adolescents, especially those in low- and middle-income countries (LMICs), are experiencing a nutritional transition in the form of a dramatic shift in food-consumption patterns from their respective countries’ traditional diet to a Westernized diet [1,2]. Fast food is a common component of Western-style diets, and is energy-dense, nutrient-poor, low in fiber and micronutrients, and high in refined grains, sodium, and sugar [3,4]. As is well known, fast-food consumption is linked to poor dietary habits (e.g. a higher intake of carbonated soft-drinks and sweets and a lower intake of fruits and vegetables) and unmet nutrient requirements [5]. Failure to meet nutrient requirements during adolescence can result in growth retardation, impaired organ remodeling, and micronutrient deficiencies [6]. Such unhealthy dietary habits in youth are thus associated with an increased risk of obesity [7], cardiovascular disease [8], type 2 diabetes [9], and cancer [10] in later adulthood.

Notably, fast-food consumption among young adolescents in LMICs has increased significantly in recent decades [11]. For example, one multinational study found that 49% of 12–15-year-old adolescents had consumed fast food at least once in the past week, while another study reported that 46.1% of adolescents consumed fast food at least once per week; however, the mean frequency (per week) of fast-food consumption and an age- and sex-specific analysis were not reported in the first study [5], and the adolescents in the second study were aged 12–17 years [12].

Therefore, our study used the latest national Global School-Based Student Health survey (GSHS) data to describe the weighted prevalence and mean frequency of fast-food consumption in 12–15-year-old adolescents by region and country in 54 LMICs during 2009–2015, and evaluated the differences between subgroups stratified by sex and age. In addition, as multiple burdens of malnutrition are common in young adolescents in LMICs [13], we also performed subgroup analyses stratified by nutritional status.

## Methods

### Data sources

We used the most recent data from the GSHS. The methods and main findings of the GSHS are described on the websites of both the U.S. Centers for Disease Control and Prevention (CDC) (https://www.cdc.gov/gshs/) [14] and the World Health Organization (WHO) (https://www.who.int/ncds/surveillance/gshs/en/) [15]. Briefly, the GSHS is a self-administered school-based survey developed by WHO and the CDC with the aim of providing data on the health behaviors of 12–15-year-old adolescents in participating countries to help those countries develop priorities, establish programs, and advocate for resources for youth health programs and policies.

The GSHS uses the same standardized sampling strategy, study methods, and questionnaire in each participating country to obtain a nationally representative sample of 12–15-year-old young adolescents. Briefly, in each country, participants are selected using a two-stage cluster procedure. In the first stage, schools are selected with a probability proportional to the size of the population, and in the second stage, classes are selected randomly from the selected schools. All of the students in the selected school classes are included in the sampling frame. If a country had participated in more than one GSHS, the latest national survey data were used in our study. Therefore, 12–15-year-old adolescents from 54 LMICs based on the latest GSHS datasets from 2009–2015 were included in our study.

The GSHS data are obtained using a self-administered questionnaire that includes 10 modules of questions that address the leading causes of morbidity and mortality among adolescents worldwide in the following areas: alcohol use, dietary behaviors, drug use, hygiene, mental health, physical activity, protective factors, sexual behaviors, tobacco use, and violence and unintentional injury. Countries are free to select different modules and translate them into their local language, but all of the questions within the selected modules must be used without modification, so that the data can be directly compared among countries. The questionnaire is anonymous and is self-administered by the students during a 40–45-min period in the classroom. The students answered all of the questions on a computer-scannable answer sheet, and the data were entered automatically at the US CDC using an automated optical-character recognition procedure. The CDC then sends an electronic database and summary results to each participating country.

All of the GSHSs were approved by both the national government’s administrative body (usually a ministry of health or education) and the institutional review board or ethics committee of each participating country. Participation was voluntary, and verbal or written consent was also obtained from the participating adolescents and their parents.

### Fast-food consumption

Fast-food consumption was assessed using the question ‘During the past 7 days, on how many days did you eat food from a fast-food restaurant?’ The response options were ‘0 days,’ ‘1 day,’ ‘2 days,’ ‘3 days,’ ‘4 days,’ ‘5 days,’ ‘6 days,’ and ‘7 days.’ The final response options were coded as 1 = 0 days/per week, 2 = 1–3 days/per week, and 3 = 4–7 days/per week. The mean frequency of fast-food consumption by adolescents (days per week) in each country was also calculated.

### Multiple burdens of malnutrition

For undernutrition, stunting was defined as height-for-age fewer than two standard deviations (SDs) below the WHO Child Growth Reference median, and thinness was defined as body mass index (BMI)-for-age fewer than two SDs below the WHO growth reference median. For overnutrition, overweight was defined as BMI-for-age greater than one SD above the WHO Growth Reference median, and obesity was defined as BMI-for-age greater than two SDs above the WHO growth reference median [13]. BMI was calculated as the weight in kilograms divided by the height in meters squared [15].

### Associated risk factors for fast-food consumption

Several potential risk factors for fast-food consumption were extracted from the data. These factors were age, sex, BMI, food insecurity, fruit consumption, vegetable consumption, soft-drink consumption, smoking, physical active, and sedentary behavior. These variables and their coding are described in Supplementary Table 1.

### Statistical analysis

Frequency estimates of fast-food consumption were based on individual data in each survey. The prevalence and mean frequency of fast-food consumption are reported as weighted estimates and 95% confidence intervals using the Statistical Analysis System (SAS) SURVEYMEANS procedure. The pooled overall and regional estimates were calculated by meta-analysis with a random-effects model using STATA [12]. Heterogeneity was assessed using I2 statistics. Subgroup analyses were performed on subgroups stratified by sex (boys vs. girls), age (12–13 years vs. 14–15 years), and nutritional status [undernutrition (stunting and thinness) vs. overnutrition (overweight and obesity)]. The differences in the prevalence of fast-food consumption between sex, age, and nutritional status subgroups were estimated using the χ^2^ test following the SAS FREQ procedure. Logistic regression analysis was used to explore the associated risk factors of fast-food consumption.

Our analysis added weights, stratum, and a primary sampling unit (PSU) to each student record in the GSHS data file to reflect the weighting process and the two-staged sampling design. The data analysis accounted for the complex sampling design uses in the GSHS. All of the data were weighted according to a random-cluster sampling design to provide nationally representative estimates for each country. The stratum reflects the first stage of the GSHS sampling (school level), and the PSU reflects the second stage (classroom level). All of the statistical analyses were performed using SAS version 9.4 (SAS Institute, Cary, NC) and STATA version 12.0 (STATA Corporation; College Station, TX). *P* values less than 0.05 were considered to indicate statistically significant differences.

## Results

Supplementary Figure 1 presents the selection process of LMICs based on the latest GSHS national survey data. Ninety-four LMICs participated in the GSHSs during 2003–2015, but 40 (43%) were excluded from our study because they lacked data on height, weight, and fast-food consumption or national data. The remaining 54 LMICs from five WHO regions included in this study were: eight (47%) from Africa, 17 (57%) from the Americas, 12 (67%) from the Eastern Mediterranean, 4 (57%) from Southeast Asia, and 13 (65%) from the Western Pacific region.

Table 1 shows the characteristics of the 54 LMICs included in this study; 153,496 young adolescents (46.09% boys) aged 12–15 years with complete data on sex, age, height, weight, and fast-food consumption during 2009–2015 were included in our study. The sample sizes ranged from 679 young adolescents in Mozambique to 21,626 young adolescents in Argentina. The average response rate was 99.32%, and it ranged from 97.39% in Qatar and Afghanistan to 99.94% in Belize and Philippines.

**Table 1.** Survey characteristics of the global school-based student health surveys (2009–2015).

	Survey year	Sample size	Response rate, %	Boys, %
**Africa Region**				
Algeria	2011	3517/3527	99.72	45.74
Benin	2009	1177/1179	99.83	63.38
Mauritania	2010	1304/1320	98.79	46.23
Mauritius	2011	2055/2075	99.04	46.05
Mozambique	2015	679/697	97.42	50.77
Namibia	2013	1977/1981	99.80	41.88
Seychelles	2015	2044/2063	99.08	46.79
United Republic of Tanzania	2014	2628/2643	99.43	44.84
**Americas Region**				
Antigua and Barbuda	2009	1228/1243	98.79	46.63
Argentina	2012	21626/21762	99.38	46.87
Barbados	2011	1490/1507	98.87	46.37
Belize	2009	1620/1621	99.94	46.87
Bolivia	2012	2927/2956	99.02	49.61
Chile	2013	1344/1354	99.26	49.02
Bahamas	2013	1297/1312	98.86	45.66
Costa Rica	2009	2270/2277	99.69	47.57
Curaçao	2015	1501/1508	99.54	46.85
El Salvador	2013	1632/1644	99.27	52.74
Guatemala	2015	3631/3666	99.05	47.97
Guyana	2010	1973/1985	99.40	44.67
Honduras	2012	1481/1502	98.60	47.37
Peru	2010	2370/2374	99.83	48.49
Suriname	2009	1061/1062	99.91	46.88
Trinidad and Tobago	2011	2375/2383	99.66	54.78
Uruguay	2012	2893/2905	99.59	47.02
**Eastern Mediterranean Region**				
Afghanistan	2014	1490/1530	97.39	37.13
Egypt	2011	2380/2424	98.18	45.96
Iraq	2012	1538/1553	99.03	54.69
Kuwait	2015	2048/2066	99.13	45.74
Lebanon	2011	1983/1995	99.40	46.44
Morocco	2010	2440/2451	99.55	50.00
Oman	2015	1673/1681	99.52	44.27
Pakistan	2009	4988/5005	99.66	74.86
Qatar	2011	1755/1802	97.39	44.55
Sudan	2012	1449/1476	98.17	35.60
Syrian Arab Republic	2010	2914/2941	99.08	39.95
United Arab Emirates	2010	2305/2313	99.65	39.07
**Southeast Asia Region**				
Bangladesh	2014	2720/2760	98.55	38.42
Indonesia	2015	8766/8824	99.34	46.24
Thailand	2015	4109/4149	99.04	47.04
Timor-Leste	2015	1668/1705	97.83	41.76
**Western Pacific Region**				
Brunei Darussalam	2014	1809/1827	99.01	46.71
Cambodia	2013	1811/1820	99.51	44.20
Kiribati	2011	1352/1358	99.56	41.84
Lao People’s Democratic Republic	2015	1658/1664	99.64	41.95
Malaysia	2012	16265/16287	99.86	51.25
Mongolia	2013	3691/3720	99.22	47.77
Philippines	2015	6163/6167	99.94	43.59
Samoa	2011	2194/2213	99.14	40.74
Solomon Islands	2011	957/979	97.75	50.33
Tonga	2010	1955/1958	99.85	44.54
Vanuatu	2011	858/865	99.19	41.58
Vietnam	2013	1738/1749	99.37	46.60
Wallis and Futuna	2015	719/725	99.17	49.30

Table 2 presents data on the prevalence of fast-food consumption over the past 7 days by region. Overall, the prevalence of fast-food consumption for 0 days, at least 1 day, 1–3 days, and 4–7 days per week were 44.1% (40.1–48.0%), 55.2% (51.3–59.1%), 45.0% (42.1–47.9%), and 10.3% (8.3–12.4%), respectively. At the region level, the prevalence of fast-food consumption for 4–7 days per week was lowest in the Americas at 8.3% (6.7–9.9%) and highest in Southeast Asia at 17.7% (2.3–33.2%). The overall frequency of fast-food consumption in young adolescents was 2.27 (2.15–2.38) times per week, with the lowest frequency of 2.19 (2.02–2.37) times per week in the Americas and the highest frequency of 2.75 (1.76–3.74) times per week in Southeast Asia (Table 2).

**Table 2.** The prevalence of fast-food consumption among adolescents aged 12–15 years, by region.

	0 days, %	1–3 days, %	4–7 days, %	At least 1 days per week, %	Mean frequency per week,
Region	(95%CI)	(95%CI)	(95%CI)	(95%CI)	(95%CI)
Africa Region	44.3	42.6	13.0	53.8	2.41
	(36.6–52.1)	(36.1–49.1)	(9.2–16.7)	(46.5–61.1)	(2.14–2.67)
Americas Region	44.6	47.0	8.3	55.3	2.19
	(38.0–51.1)	(42.0–52.0)	(6.7–9.9)	(48.8–61.8)	(2.02–2.37)
Eastern Mediterranean Region	43.2	46.5	10.2	56.8	2.23
	(30.8–55.7)	(38.2–54.7)	(6.9–13.5)	(44.5–69.1)	(1.93–2.54)
Southeast Asia Region	36.1	46.1	17.7	56.8	2.75
	(21.4–50.8)	(37.6–54.6)	(2.3–33.2)	(44.5–69.1)	(1.76–3.74)
Western Pacific Region	46.1	42.6	11.0	53.1	2.29
	(37.2–55.1)	(36.2–49.0)	(7.8–14.1)	(43.7–62.6)	(2.01–2.57)
Total	44.1	45.0	10.3	55.2	2.27
	(40.1–48.0)	(42.1–47.9)	(8.3–12.4)	(51.3–59.1)	(2.15–2.38)

**Figure 1.**
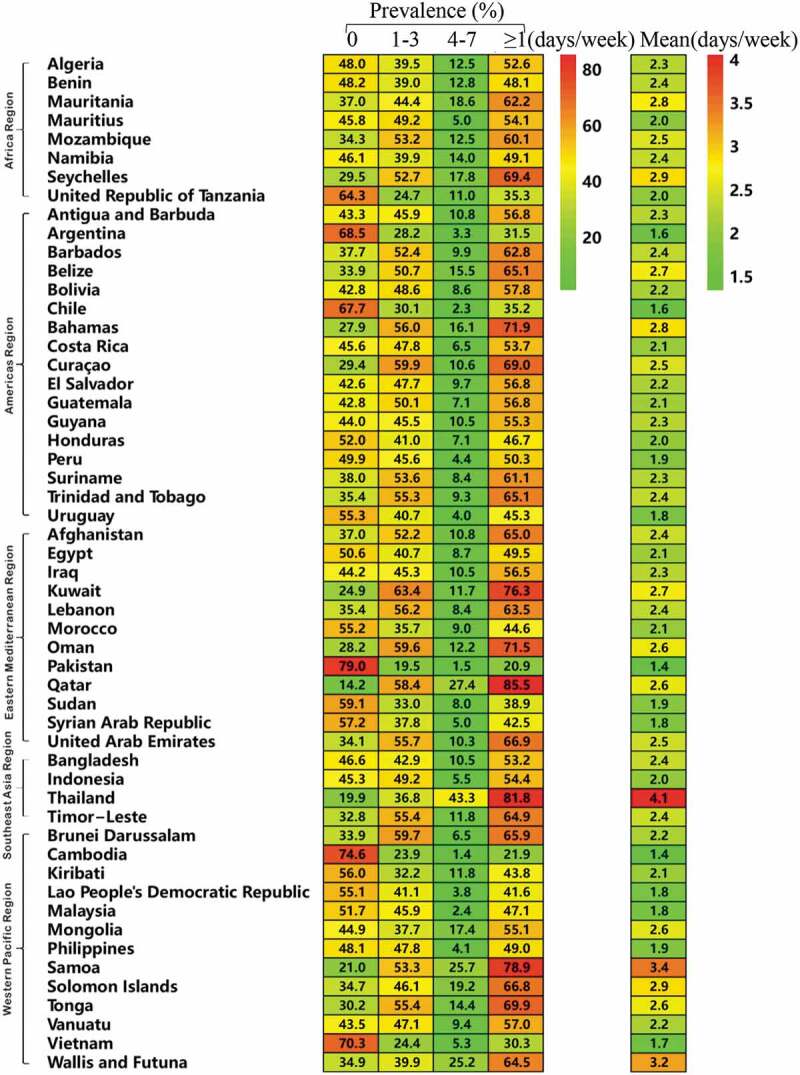
Frequency of fast-food consumption among adolescents aged 12–15 years, by country.

As shown in Figure 1 and Supplementary Table 2, at the country level, the prevalence of fast-food consumption for 4–7 days per week was lowest in Pakistan at 1.5% (1.0–2.0%) and highest in Thailand at 43.3% (40.4–46.1%). The overall mean frequency of fast-food consumption was also lowest in Pakistan at 1.35 (1.28–1.41) times per week and highest in Thailand at 4.06 (3.92–4.19) times per week.

Supplementary Tables 3–5 present data on the prevalence of fast-food consumption by subgroups. Overall, the prevalence of fast food consumption for 4–7 days per week was not different between boys and girls [10.7% (8.1–13.4) vs. 10.0% (8.3–11.7%), *P* = 0.058] or between the 12–13-year age group and the 14–15-year age group [10.3% (8.1–12.6%) vs. 10.3% (8.3–12.3%), *P* = 0.999] (Supplementary Tables 3 and 4). Furthermore, as for the subgroups stratified by nutritional status (undernutrition and overnutrition), the prevalence of fast-food consumption for 4–7 days per week was 7.6% (4.6–10.5%) and 7.7% (5.9–9.5%) in the stunting and thinness groups, respectively, and 8.6% (6.4–10.9%) and 6.6% (4.5–8.7%) in the overweight and obesity groups, respectively, indicating that the prevalence was lowest in the obesity group; however, combined with confidence intervals, this result was not statistically significant (Supplementary Table 5).

Age and BMI were found to be negatively correlated with fast-food consumption [odds ratio: 0.937 (0.920–0.954) and 0.994 (0.990–0.999)], whereas sex, food insecurity, fruit consumption, vegetable consumption, soft-drink consumption, smoking, physical activity, and sedentary behavior [odds ratio: 1.081 (1.043–1.120), 1.535 (1.385–1.702), 1.208 (1.164–1.253), 1.517 (1.458–1.578), 2.254 (2.173–2.338), 1.491 (1.362–1.632), 1.258 (1.209–1.308), and 1.491 (1.439–1.545)] were positively correlated with fast-food consumption (Table 3).

**Table 3.** Association risk factors for fast-food consumption in five regions and total.

	Africa Region	Americas Region	Eastern Mediterranean Region	Southeast Asia Region	Western Pacific Region	Total
Age	1.061	0.917	1.004	1.113	0.856	0.937
	(1.008–1.116)	(0.880–0.954)	(0.959–1.052)	(1.069–1.159)	(0.824–0.888)	(0.920–0.954)
Sex	0.712	1.121	1.076	1.220	1.064	1.081
	(0.641–0.792)	(1.041–1.207)	(0.986–1.174)	(1.123–1.326)	(0.993–1.140)	(1.043–1.120)
BMI	0.975	0.983	1.019	1.002	1.028	0.994
	(0.961–0.989)	(0.974–0.992)	(1.010–1.029)	(0.991–1.013)	(1.021–1.035)	(0.990–0.999)
Food insecurity	1.252	2.438	1.218	0.921	1.602	1.535
	(0.971–1.614)	(1.972–3.014)	(0.939–1.581)	(0.700–1.211)	(1.317–1.948)	(1.385–1.702)
Fruits consumption	1.599	1.154	1.222	1.585	1.007	1.208
	(1.432–1.786)	(1.069–1.245)	(1.115–1.339)	(1.454–1.728)	(0.937–1.081)	(1.164–1.253)
Vegetables consumption	1.299	1.431	1.233	1.454	1.303	1.517
	(1.160–1.455)	(1.305–1.569)	(1.113–1.367)	(1.332–1.588)	(1.209–1.404)	(1.458–1.578)
Soft Drinking consumption	2.066	2.655	3.354	2.355	2.377	2.254
	(1.837–2.322)	(2.420–2.913)	(3.064–3.670)	(2.169–2.559)	(2.218–2.547)	(2.173–2.338)
Smoking	1.667	1.429	1.664	0.943	1.762	1.491
	(1.301–2.135)	(1.192–1.714)	(1.346–2.056)	(0.725–1.226)	(1.488–2.087)	(1.362–1.632)
Physical active	1.151	1.065	1.283	1.493	1.484	1.258
	(1.022–1.298)	(0.982–1.154)	(1.156–1.424)	(1.361–1.638)	(1.374–1.602)	(1.209–1.308)
Sedentary behavior	1.580	1.284	2.178	2.096	1.432	1.491
	(1.421–1.757)	(1.193–1.382)	(1.994–2.378)	(1.926–2.282)	(1.336–1.534)	(1.439–1.545)

## Discussion

Our study revealed a high overall prevalence of fast-food consumption among 153,496 young adolescents in 54 LMICs. Approximately 55.2% of adolescents consumed fast food at least 1 day per week, and the overall mean frequency of fast-food consumption was 2.3 times per week.

The overall prevalence of fast-food consumption for at least 1 day per week found in our study was much higher than that reported in the literature (55.2% vs. 49.1%) [5,12]. Only adolescents with complete data on height, weight, and fast-food consumption were included in our study, therefore the prevalence obtained in our study may be more realistic and reliable. Globalization and modernization have increased the accessibility of fast-food chain restaurants in LMICs [16]. Currently, McDonald’s, Kentucky Fried Chicken (KFC), and Pizza Hut are dominant fast-food chains in these LMICs [16]. Food environments are shifting rapidly in many LMICs, leading to changes in food consumption and dietary habits. Healthy-diet promotion programs and policies targeted at young adolescents should be implemented in LMICs, and their effectiveness should be evaluated.

Notably, at the regional level, the overall prevalence of fast-food consumption for 4–7 days per week in young adolescents was found to be highest in Southeast Asia (17.7%). This is consistent with the fact that the socioeconomic status (SES) of Asian countries has improved greatly, which has increased their populations’ accessibility to unhealthy energy-rich fast foods and the prevalence of a more mechanized, more sedentary, and less labor-intensive daily routine [16]. In our study, sedentary behavior in young adolescents also had a positive correlation with fast-food consumption, which is in agreement with the fact that with the technological advancement in Asian countries, television programs have started occupying most of the leisure time of adolescents [16]. As is known, some television programs create an image of fast-food health, power, and fashion via endorsements by popular entertainment stars and sports stars to lure adolescents into eating high-calorie foods which, in excess, are unhealthy [17–19]. In addition, food industries have invested millions of dollars in popularizing Western-style fast food in Asian countries, and governments in the region have done little to promote healthier food options [17].

Only four Asian countries were included in our study, of which Thailand had the highest prevalence of fast-food consumption for 4–7 days per week among young adolescents. This can be partly explained by the high prevalence of fast-food chain restaurants in Asia. Thailand, as a middle-income country, has been considered a new market for penetration by the processed food industry [20], which provides a greater supply of and increases the demand for fast foods and beverages [21]. Indeed, the traditional Thai food culture has changed dramatically, and Thais now follow fashion/trends in food consumption [2]. Western-style fast-food consumption is playing an increasingly important role in the Thai food culture [2]. Therefore, although key actions have been taken by the Thai government, such as nutritional labelling, providing nutrition claims [20], and the use of Guideline Daily Amounts [20,21], these actions appear to be inadequate.

In contrast, the Americas (8.3%) showed the lowest prevalence of fast-food consumption for 4–7 days per week. Notably, all Latin American countries signed the Plan of Action for the Prevention of Obesity in Children and Adolescents in 2014 [22]. Under this plan, these countries have taken a series of measures to improve the food environment [22], such as (i) implementing new policies to improve school nutrition, including nutritional guidelines for school meals and school meal programs (currently, approximately 85 million students receive daily school meals in the Latin American region); (ii) regulating food marketing to children and adolescents; and (iii) introducing mandatory and voluntary nutrition labeling, such as labels on both the back and front of food packages. These measures may have contributed to the lowest prevalence of fast-food consumption observed in this region.

Another interesting finding of this study was that Pakistan (1.5%) had the lowest prevalence of fast-food consumption for 4–7 days per week among adolescents. Pakistan is one of the first developing countries to formulate a comprehensive National Action Plan for Non-Communicable Disease Prevention, Control and Health Promotion [23]. The actions in this plan comprised revision of the current policy on diet and nutrition; strategies to limit the production of and access to ghee as a medium for cooking; and agricultural and fiscal policies that aimed to increase the accessibility to more healthy foods [24]. In 2013, Pakistan also joined the ‘Scaling Up Nutrition Movement’ to show its commitment to develop culturally tailored dietary guidelines [25]. These actions may have contributed to the lowest prevalence of fast-food consumption in Pakistan.

The prevalence of fast-food consumption for 4–7 days per week was lowest in the obesity group, although the result was not statistically significant. Consistent with our findings, another research also found that obesity was not associated with fast-food consumption among adolescents [26]. In our study, height and weight in the GSHSs were self-reported [27]; thus, the prevalence of obesity might be underestimated. Previous studies have shown that SES was negatively associated with fast-food consumption [28,29]. Our detailed logistic-regression analysis revealed that food insecurity, which is usually regarded as a proxy for SES, was positively associated with fast-food consumption (Table 3). All of these findings indicate that the confounding factors, such as SES, should be considered in the analysis between obesity and fast food consumption.

Unhealthy eating patterns formed in adolescence have lasting effects on future health [12]. Considering the popularity of fast food among young adolescents in LMICs, there is an urgent need for policies, strategies, and recommendations to develop a healthy dietary environment in these countries. Examples include marketing controls, including the enaction and enforcement of regulations to limit the advertisement and sale of fast food [30], attaching front-of-pack supplementary nutritional labels, and implementing formula fast-foods contain less salt, saturated fat, trans-fat, sugar, and energy [30]; implementing fiscal policies (e.g. increasing the relative price of fast food by imposing taxes and then using these taxes to subsidize healthy food products) [31]; strengthening the cooperation of schools and families, providing nutrition knowledge and health education, limiting the number of fast-food outlets in schools and neighborhoods, teaching traditional cooking skills, and advocating for the nutritional worth of foods [32]. Furthermore, nutrition guidelines specifically for young adolescents should be promoted globally, especially in LMICs, in accordance with their social, cultural, and economic backgrounds.

To the best of our knowledge, this is the first study to comprehensively estimate the prevalence of fast-food consumption among young adolescents stratified by age, sex, and nutritional status. The strengths of this study also include the timely and comprehensive assessment of fast-food consumption (days per week/times per week), large sample size, standardized procedures for the selection of participants, direct comparisons, and prevalence estimation with respect to the study design. Furthermore, we used the latest national GSHS data from 2009 to 2015, which had a response rate of 99.32%, and only adolescents with complete data on height, weight, and fast-food consumption were included, indicating more reliable findings.

However, the following limitations should be acknowledged. First, the GSHSs were mainly conducted in schools; thus, the results might not sufficiently reflect the prevalence of fast-food consumption among all adolescents in LMICs. More specifically, the prevalence of fast-food consumption among adolescents who are unable to attend schools or have dropped out could not be acquired. Second, variables in the GSHSs were self-reported by adolescents; therefore, the possibility of recall bias cannot be ruled out. Third, the surveys included in the cross-sectional analysis were conducted over a long period (2009–2015); therefore, direct comparisons between countries should be made cautiously. Fourth, the amounts of fast food consumed each time were not available; thus, the prevalence of fast-food consumption might have been underestimated. Finally, we observed high heterogeneity in the meta-analyses when pooling estimates across countries. However, heterogeneity can be overestimated when summarizing studies with large sample sizes [33].

In conclusion, the overall prevalence of fast-food consumption was consistently high among 12–15-year-old young adolescents in LMICs. Therefore, there is an urgent need to prioritize the implementation of healthy-diet promotion programs targeting this population.

## Supplementary Material

Supplemental Material

## Paper context

The prevalence of fast-food consumption worldwide has increased significantly over the past decade, particularly in LMICs. In this paper, we described the overall prevalence of fast-food consumption among young adolescents in LMICs and the differences between subgroups stratified by sex, age, and nutritional status. There is an urgent need to prioritize the implementation of healthy-diet promotion programs targeting this population.
