# Supplementary material for: Fast food consumption among young adolescents aged 12–15 years in 54 low- and middle-income countries
Source: Glob Health Action. 2020 Aug 7;13(1):1795438. doi: 10.1080/16549716.2020.1795438 (PMC7480506; doi:10.1080/16549716.2020.1795438)
Supplement: Supplemental Material [file ZGHA_A_1795438_SM4883.docx]

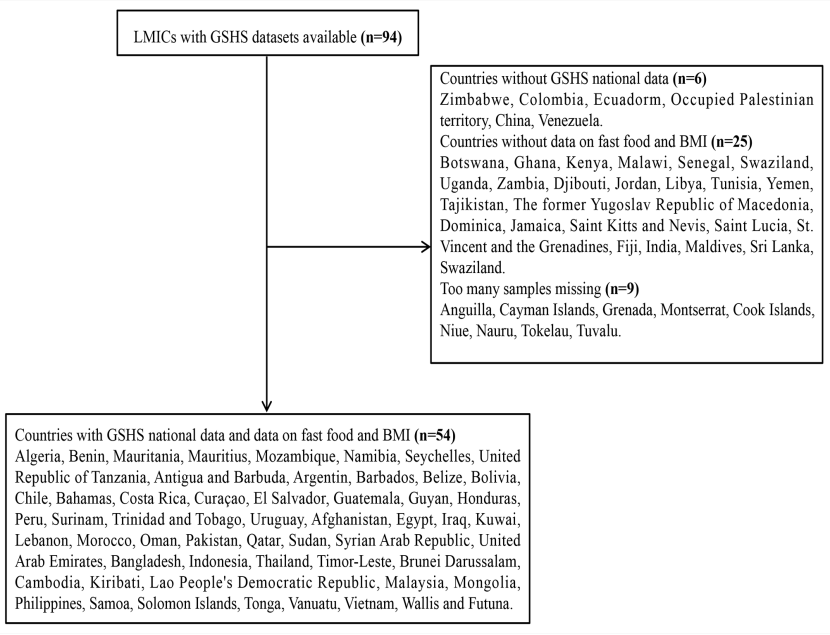


**Supplementary Figure 1.** Selection process for low-income and middle-income countries (LMICs) using the most recent Global School-based Student Health Survey (GSHS) national data.

**Supplementary Table 1.** Independent variable derivation from survey data.

| Survey question | Coding | Variable |
| --- | --- | --- |
| During the past 7 days, on how many days did you eat food from a fast food restaurant, such as [country specific examples]? | 3+ days code as 1;  Less than 3 days code as 0. | Fast food consumption |
| How old are you? | 12-15 years | Age |
| What is your sex? | Girls code as 1; Boys code as 0. | Sex |
| How tall are you without your shoes on? | BMI=weight/height^2^ | BMI |
| How much do your weight without your shoes on? |  |  |
| During the past 30 days, how often did you go hungry because there was not enough food in your home? | Most of the time/always code as 1; Never/rarely/sometimes code as 0. | Food insecurity |
| During the past 30 days, how many times per day did you usually eat fruit? (examples of fruit provided) | 2+ times a day code as 1;  Less than 2 times a day code as 0. | Fruits consumption |
| During the past 30 days, how many times per day did you usually eat vegetables? (examples of vegetables provided) | 3+ times a day code as 1；  Less than 3 times a day code as 0. | Vegetables consumption |
| During the past 30 days, how many times per day did you usually drink carbonated soft drinks, such as Coca-Cola, Sprite, Fanta, Fizzi, Moka, Youki, or tonic? | 1+ times a day code as 1；  Less than 1 per day code as 0. | Soft drinking consumption |
| How old were you when you first tried a cigarette? | Never smoked cigarettes code as 1；Other answers code as 0. | Smoking |
| During the past 7 days, on how many days were you physically active for a total of at least 60 minutes per day? | 5+ days code as 1；Less than 5 days code as 0. | Physical active |
| How much time do you spend during a typical or usual day sitting and watching television, playing computer games, talking with friends, or doing other sitting activities such as [country specific examples]? | 3+ hours per day code as 1；  Less than 3 hours per day code as 0. | Sedentary behavior |

**Supplementary Table 2.** The weighted prevalence of Fast-Food Consumption Among Adolescents Aged 12–15 Years, by Region and Country.

|  | **Total, % (95%CI)** | | | |  |
| --- | --- | --- | --- | --- | --- |
|  | **0 days** | **1-3 days** | **4-7 days** | **at least**  **1 days**  **per week** | **Mean frequency per week, (95%CI)** |
| **Africa Region** | | | | | |
| Algeria | 48.0  (44.7-51.3) | 39.5  (36.7-42.2) | 12.5  (10.5-14.6) | 52.6  (48.9-56.3) | 2.32  (2.20-2.45) |
| Benin | 48.2  (44.5-51.9) | 39.0  (36.2-41.8) | 12.8  (10.2-15.4) | 48.1  (44.1-52.0) | 2.36  (2.18-2.55) |
| Mauritania | 37.0  (31.0-43.1) | 44.4  (40.6-48.2) | 18.6  (15.4-21.8) | 62.2  (56.6-67.8) | 2.78  (2.56-3.00) |
| Mauritius | 45.8  (43.5-51.4) | 49.2  (47.0-51.4) | 5.0  (3.6-6.3) | 54.1  (51.9-56.4) | 1.97  (1.89-2.04) |
| Mozambique | 34.3  (23.4-45.3) | 53.2  (43.9-62.5) | 12.5  (9.1-15.9) | 60.1  (50.1-70.1) | 2.55  (2.23-2.86) |
| Namibia | 46.1  (40.2-52.0) | 39.9  (35.7-44.0) | 14.0  (10.8-17.3) | 49.1  (44.4-53.8) | 2.41  (2.19-2.64) |
| Seychelles | 29.5  (26.6-32.4) | 52.7  (50.1-55.2) | 17.8  (15.0-20.7) | 69.4  (66.7-72.1) | 2.87  (2.72-3.01) |
| United Republic of Tanzania | 64.3  (59.1-69.5) | 24.7  (21.6-27.8) | 11.0  (7.8-14.2) | 35.3  (30.4-40.2) | 2.03  (1.82-2.25) |
| Pooled estimates | 44.3  (36.6-52.1) | 42.6  (36.1-49.1) | 13.0  (9.2-16.7) | 53.8  (46.5-61.1) | 2.41  (2.14-2.67) |
| I^2^ (%) | 96.0 | 97.1 | 94.4 | 96.5 | 95.6 |
| **Americas Region** | | | | | |
| Antigua and Barbuda | 43.3  (39.6-47.0) | 45.9  (42.3-49.6) | 10.8  (7.7-13.8) | 56.8  (53.2-60.4) | 2.31  (2.14-2.49) |
| Argentina | 68.5  (66.5-70.5) | 28.2  (26.3-30.1) | 3.3  (2.7-3.9) | 31.5  (29.7-33.3) | 1.60  (1.55-1.65) |
| Barbados | 37.7  (34.5-40.9) | 52.4  (49.4-55.3) | 9.9  (8.2-11.7) | 62.8  (59.7-65.9) | 2.37  (2.26-2.48) |
| Belize | 33.9  (28.9-38.9) | 50.7  (46.7-54.6) | 15.5  (12.3-18.7) | 65.1  (60.1-70.1) | 2.70  (2.51-2.88) |
| Bolivia | 42.8  (40.5-45.1) | 48.6  (46.2-50.9) | 8.6  (7.5-9.7) | 57.8  (55.7-59.9) | 2.21  (2.14-2.27) |
| Chile | 67.7  (64.4-70.9) | 30.1  (27.1-33.0) | 2.3  (1.1-3.5) | 35.2  (32.4-38.0) | 1.55  (1.47-1.63) |
| Bahamas | 27.9  (23.5-32.2) | 56.0  (51.9-60.1) | 16.1  (13.7-18.5) | 71.9  (67.3-76.5) | 2.84  (2.69-3.00) |
| Costa Rica | 45.6  (40.1-51.1) | 47.8  (43.1-52.6) | 6.5  (5.1-7.9) | 53.7  (48.7-58.7) | 2.08  (1.95-2.21) |
| Curaçao | 29.4  (26.9-31.9) | 59.9  (57.7-62.1) | 10.6  (9.1-12.1) | 69.0  (66.8-71.1) | 2.54  (2.44-2.64) |
| El Salvador | 42.6  (39.2-45.9) | 47.7  (44.8-50.7) | 9.7  (7.6-11.8) | 56.8  (53.4-60.2) | 2.24  (2.12-2.37) |
| Guatemala | 42.8  (35.3-50.3) | 50.1  (43.9-56.3) | 7.1  (5.1-9.0) | 56.8  (49.5-64.1) | 2.14  (1.93-2.35) |
| Guyana | 44.0  (41.1-46.9) | 45.5  (42.9-48.1) | 10.5  (8.4-12.5) | 55.3  (52.5-58.1) | 2.32  (2.19-2.44) |
| Honduras | 52.0  (48.3-55.6) | 41.0  (37.4-44.6) | 7.1  (5.9-8.2) | 46.7  (43.1-50.2) | 2.05  (1.95-2.14) |
| Peru | 49.9  (45.9-54.0) | 45.6  (41.8-49.5) | 4.4  (3.5-5.3) | 50.3  (46.1-54.5) | 1.91  (1.83-1.99) |
| Suriname | 38.0  (33.3-42.7) | 53.6  (49.2-57.9) | 8.4  (6.4-10.5) | 61.1  (56.9-65.3) | 2.29  (2.17-2.41) |
| Trinidad and Tobago | 35.4  (31.3-39.6) | 55.3  (52.3-58.2) | 9.3  (7.4-11.1) | 65.1  (60.8-69.3) | 2.38  (2.25-2.52) |
| Uruguay | 55.3  (52.4-58.2) | 40.7  (38.0-43.5) | 4.0  (3.3-4.7) | 45.3  (42.5-48.1) | 1.79  (1.73-1.85) |
| Pooled estimates | 44.6  (38.0-51.1) | 47.0  (42.0-52.0) | 8.3  (6.7-9.9) | 55.3  (48.8-61.8) | 2.19  (2.02-2.37) |
| I^2^ (%) | 98.5 | 97.8 | 96.0 | 98.6 | 98.2 |
| **Eastern Mediterranean Region** | | | | | |
| Afghanistan | 37.0  (30.4-43.5) | 52.2  (46.2-58.1) | 10.8  (8.2-13.4) | 65.0  (58.0-72.0) | 2.45  (2.26-2.64) |
| Egypt | 50.6  (44.1-57.1) | 40.7  (35.3-46.0) | 8.7  (6.0-11.4) | 49.5  (42.7-56.4) | 2.14  (1.95-2.33) |
| Iraq | 44.2  (39.7-48.7) | 45.3  (41.8-48.9) | 10.5  (8.4-12.6) | 56.5  (52.0-60.9) | 2.32  (2.17-2.46) |
| Kuwait | 24.9  (19.2-30.6) | 63.4  (59.2-67.6) | 11.7  (9.1-14.2) | 76.3  (72.5-80.2) | 2.70  (2.49-2.92) |
| Lebanon | 35.4  (32.2-38.7) | 56.2  (52.6-59.8) | 8.4  (5.9-10.9) | 63.5  (60.2-66.8) | 2.37  (2.24-2.49) |
| Morocco | 55.2  (51.1-59.4) | 35.7  (33.0-38.5) | 9.0  (6.6-11.5) | 44.6  (40.5-48.8) | 2.06  (1.91-2.21) |
| Oman | 28.2  (24.6-31.8) | 59.6  (57.0-62.2) | 12.2  (9.9-14.5) | 71.5  (68.1-74.8) | 2.64  (2.47-2.81) |
| Pakistan | 79.0  (75.6-82.5) | 19.5  (16.3-22.7) | 1.5  (1.0-2.0) | 20.9  (17.5-24.3) | 1.35  (1.28-1.41) |
| Qatar | 14.2  (11.9-16.6) | 58.4  (54.7-62.1) | 27.4  (24.0-30.7) | 85.5  (83.2-87.9) | 2.62  (2.47-3.77) |
| Sudan | 59.1  (53.0-65.2) | 33.0  (28.1-37.9) | 8.0  (5.3-10.6) | 38.9  (33.4-44.4) | 1.94  (1.75-2.13) |
| Syrian Arab Republic | 57.2  (51.9-62.6) | 37.8  (32.9-42.7) | 5.0  (3.9-6.0) | 42.5  (37.1-47.8) | 1.85  (1.73-1.96) |
| United Arab Emirates | 34.1  (29.2-39.0) | 55.7  (51.5-59.8) | 10.3  (8.6-11.9) | 66.9  (62.1-71.6) | 2.47  (2.33-2.61) |
| Pooled estimates | 43.2  (30.8-55.7） | 46.5  (38.2-54.7) | 10.2  (6.9-13.5) | 56.8  (44.5-69.1) | 2.23  (1.93-2.54) |
| I^2^ (%) | 99.1 | 98.3 | 97.9 | 99.1 | 98.2 |
| **Southeast Asia Region** | | | | | |
| Bangladesh | 46.6  (41.8-51.4) | 42.9  (38.2-47.6) | 10.5  (7.8-13.2) | 53.2  (48.2-58.2) | 2.43  (2.24-2.63) |
| Indonesia | 45.3  (42.8-47.8) | 49.2  (47.0-51.4) | 5.5  (4.9-6.0) | 54.4  (52.3-56.6) | 2.05  (1.98-2.11) |
| Thailand | 19.9  (18.1-21.7) | 36.8  (34.6-39.0) | 43.3  (40.4-46.1) | 81.8  (80-83.5) | 4.06  (3.92-4.19) |
| Timor-Leste | 32.8  (29.0-36.6) | 55.4  (52.2-58.5) | 11.8  (8.9-14.8) | 64.9  (62.1-67.8) | 2.45  (2.27-2.62) |
| Pooled estimates | 36.1  (21.4-50.8) | 46.1  (37.6-54.6) | 17.7  (2.3-33.2) | 63.6  (48.4-78.9) | 2.75  (1.76-3.74) |
| I^2^ (%) | 99.0 | 97.3 | 99.6 | 99.3 | 99.6 |
| **Western Pacific Region** | | | | | |
| Brunei Darussalam | 33.9  (31.6-36.2) | 59.7  (57.0-62.4) | 6.5  (5.2-7.7) | 65.9  (64.1-67.7) | 2.25  (2.20-2.31) |
| Cambodia | 74.6  (71.4-77.8) | 23.9  (21.1-26.8) | 1.4  (0.6-2.3) | 21.9  (19.2-24.5) | 1.41  (1.33-1.49) |
| Kiribati | 56.0  (51.7-60.2) | 32.2  (29.2-35.2) | 11.8  (9.6-14.0) | 43.8  (39.8-47.8) | 2.11  (1.97-2.26) |
| Lao People's  Democratic Republic | 55.1  (49.4-60.9) | 41.1  (35.8-46.4) | 3.8  (2.6-4.9) | 41.6  (36.8-46.5) | 1.80  (1.69-1.92) |
| Malaysia | 51.7  (50.0-53.3) | 45.9  (44.3-47.5) | 2.4  (2.1-2.8) | 47.1  (45.7-48.4) | 1.75  (1.71-1.78) |
| Mongolia | 44.9  (39.4-50.4) | 37.7  (35.4-40.1) | 17.4  (13.2-21.5) | 55.1  (49.6-60.7) | 2.59  (2.34-2.84) |
| Philippines | 48.1  (42.2-54.0) | 47.8  (42.8-52.9) | 4.1  (2.6-5.5) | 49.0  (43.6-54.3) | 1.89  (1.75-2.03) |
| Samoa | 21.0  (17.6-24.5) | 53.3  (49.7-56.9) | 25.7  (23.6-27.8) | 78.9  (75.5-82.2) | 3.43  (3.30-3.56) |
| Solomon Islands | 34.7  (24.9-44.4) | 46.1  (39.4-52.7) | 19.2  (13.6-24.9) | 66.8  (58.1-75.4) | 2.87  (2.50-3.23) |
| Tonga | 30.2  (27.5-32.9) | 55.4  (52.8-58.0) | 14.4  (12.1-16.6) | 69.9  (67.3-72.5) | 2.65  (2.54-2.77) |
| Vanuatu | 43.5  (32.8-54.2) | 47.1  (36.7-57.5) | 9.4  (6.6-12.2) | 57.0  (47.2-66.7) | 2.22  (1.96-2.45) |
| Vietnam | 70.3  (66.3-74.3) | 24.4  (21.1-27.7) | 5.3  (3.1-7.4) | 30.3  (27.7-32.9) | 1.66  (1.52-1.81) |
| Wallis and Futuna | 34.9  (31.0-38.9) | 39.9  (36.2-43.5) | 25.2  (21.3-29.1) | 64.5  (60.9-68.1) | 3.21  (2.99-3.44) |
| Pooled estimates | 46.1  (37.2-55.1） | 42.6  (36.2-49.0) | 11.0  (7.8-14.1) | 53.1  (43.7-62.6) | 2.29  (2.01-2.57) |
| I^2^ (%) | 98.8 | 98.2 | 98.6 | 99.2 | 99.0 |
| **Total** | | | | | |
| Pooled estimates | 44.1  (40.1-48.0) | 45.0  (42.1-47.9) | 10.3  (8.3-12.4) | 55.2  (51.3-59.1) | 2.27  (2.15-2.38) |
| I^2^(%) | 0.0 | 0.0 | 47.6 | 0.0 | 0.0 |

**Supplementary Table 3.** The weighted prevalence of Fast-Food Consumption Among Adolescents Aged 12–15 Years, by Region, Country and Sex.

|  | **Boys, % (95%CI)** | | | **Girls, %(95%CI)** | | |
| --- | --- | --- | --- | --- | --- | --- |
|  | **0 days** | **1-3 days** | **4-7 days** | **0 days** | **1-3 days** | **4-7 days** |
| **Africa Region** | | | | | | |
| Algeria | 37.2  (33.3-41.1) | 44.9  (40.8-48.9) | 18.0  (14.6-21.3) | 56.9  (52.9-60.9) | 35.0  (30.9-39.2) | 8.1  (5.7-10.5) |
| Benin | 47.5  (42.0-53.1) | 38.8  (34.9-42.6) | 13.7  (10.3-17.0) | 49.7  (45.2-54.1) | 39.6  (34.6-44.5) | 10.8  (6.6-15.0) |
| Mauritania | 41.6  (35.8-47.3) | 40.5  (35.5-45.6) | 17.9  (15.4-20.4) | 31.1  (23.8-38.3) | 49.4  (45.2-53.6) | 19.5  (14.2-24.8) |
| Mauritius | 46.3  (42.9-49.8) | 47.8  (45.3-50.4) | 5.8  (3.6-8.1) | 45.2  (40.8-49.7) | 50.5  (45.7-55.3) | 4.3  (3.2-5.3) |
| Mozambique | 38.7  (31.7-45.7) | 47.3  (41.6-53.0) | 14.0  (10.3-17.7) | 30.7  (19.9-41.5) | 57.7  (46.7-68.6) | 11.6  (7.6-15.6) |
| Namibia | 42.5  (36.1-49.0) | 42.1  (37.3-47.0) | 15.3  (11.9-18.7) | 48.7  (42.3-55.1) | 38.1  (33.9-42.4) | 13.2  (9.3-17.0) |
| Seychelles | 29.7  (25.9-33.6) | 51.3  (47.8-54.8) | 19.0  (15.6-22.4) | 29.2  (26.1-32.3) | 54.0  (50.9-57.2) | 16.7  (13.4-20.0) |
| United Republic of Tanzania | 63.6  (57.1-70.0) | 26.1  (22.0-30.3) | 10.3  (6.7-13.9) | 64.3  (59.2-69.5) | 23.7  (20.4-27.0) | 11.9  (8.3-15.5) |
| Pooled estimates | 43.2  (36.7-49.8) | 42.4  (36.9-47.9) | 14.2  (10.6-17.9) | 44.7  (35.1-54.3) | 43.2  (34.7-51.7) | 11.8  (7.8-15.8) |
| I^2^ (%) | 92.9 | 93.5 | 91.0 | 96.7 | 96.8 | 93.4 |
| **Americas Region** | | | | | | |
| Antigua and Barbuda | 48.4  (44.3-52.6) | 42.5  (37.8-47.1) | 9.1  (4.3-13.9) | 37.5  (32.9-42.2) | 50.0  (44.9-55.1) | 12.5  (9.3-15.6) |
| Argentina | 68.8  (65.8-71.7) | 27.6  (25.2-30.0) | 3.6  (2.5-4.8) | 68.1  (65.7-70.5) | 28.8  (26.5-31.1) | 3.1  (2.5-3.7) |
| Barbados | 40.8  (36.2-45.4) | 48.9  (44.4-53.3) | 10.4  (7.8-12.9) | 34.6  (31.4-37.8) | 56.0  (52.5-59.4) | 9.5  (7.1-11.8) |
| Belize | 34.8  (29.0-40.5) | 51.5  (46.4-56.6) | 13.8  (10.5-17.0) | 32.8  (27.8-37.8) | 50.3  (46.3-54.2) | 16.9  (12.8-21.1) |
| Bolivia | 42.6  (39.9-45.4) | 49.2  (46.4-52.1) | 8.1  (6.6-9.7) | 42.9  (39.5-46.2) | 47.8  (44.5-51.0) | 9.4  (8.0-10.7) |
| Chile | 67.8  (62.6-73.0) | 29.5  (25.0-33.9) | 2.7  (0.6-4.8) | 67.4  (62.8-72.0) | 30.6  (26.5-34.8) | 2.0  (0.6-3.3) |
| Bahamas | 29.8  (25.7-33.9) | 55.3  (51.2-59.4) | 14.9  (11.8-18.1) | 26.2  (20.0-32.3) | 56.5  (51.1-62.0) | 17.3  (14.2-20.4) |
| Costa Rica | 48.4  (42.5-54.2) | 45.1  (40.1-50.1) | 6.5  (4.1-9.0) | 43.0  (37.2-48.8) | 50.5  (45.5-55.4) | 6.6  (5.0-8.1) |
| Curaçao | 31.1  (27.5-34.8) | 58.7  (55.4-61.9) | 10.2  (8.1-12.3) | 27.3  (24.5-30.2) | 61.4  (58.4-64.4) | 11.3  (9.1-13.4) |
| El Salvador | 44.9  (41.7-48.2) | 45.9  (43.1-48.7) | 9.1  (7.6-10.7) | 39.9  (35.4-44.5) | 49.8  (45.9-53.7) | 10.3  (6.5-14.1) |
| Guatemala | 44.2  (38.2-50.3) | 49.0  (43.3-54.6) | 6.8  (5.0-8.6) | 41.0  (31.7-50.3) | 51.3  (43.3-59.3) | 7.7  (5.5-9.9) |
| Guyana | 41.4  (37.7-45.2) | 46.1  (41.1-51.2) | 12.4  (9.1-15.8) | 45.9  (41.6-50.3) | 44.9  (41.7-48.1) | 9.1  (6.8-11.5) |
| Honduras | 51.7  (45.7-57.7) | 42.1  (36.5-47.8) | 6.2  (4.7-7.7) | 52.0  (47.5-56.5) | 40.3  (35.6-44.9) | 7.8  (5.9--9.7) |
| Peru | 49.3  (45.0-53.6) | 45.8  (41.5-50.1) | 4.9  (3.8-6.0) | 50.6  (46.0-55.2) | 45.4  (41.3-49.5) | 4.0  (2.6-5.4) |
| Suriname | 37.0  (32.1-41.8) | 54.9  (49.7-60.1) | 8.2  (5.4-10.9) | 39.4  (33.3-45.4) | 52.0  (47.2-56.8) | 8.6  (6.3-10.9) |
| Trinidad and Tobago | 39.7  (35.2-44.1) | 52.8  (49.1-56.5) | 7.5  (5.7-9.3) | 31.4  (25.7-37.1) | 57.6  (53.2-62.0) | 11.0  (8.6-13.4) |
| Uruguay | 56.4  (52.7-60.1) | 39.5  (35.8-43.2) | 4.1  (2.7-5.5) | 54.5  (51.3-57.8) | 41.7  (38.5-44.9) | 3.8  (3.0-4.5) |
| Pooled estimates | 45.7  (40.1-51.4) | 46.1  (41.4-50.9) | 7.9  (6.5-9.3) | 43.3  (36.3-50.3) | 47.9  (42.8-53.0) | 8.6  (6.8-10.4) |
| I^2^ (%) | 96.9 | 96.0 | 89.7 | 98.0 | 96.7 | 95.3 |
| **Eastern Mediterranean Region** | | | | | | |
| Afghanistan | 32.3  (21.8-42.7) | 56.0  (47.1-64.9) | 11.7  (7.2-16.3) | 42.4  (35.7-49.1) | 47.8  (42.1-53.6) | 9.8  (7.4-12.1) |
| Egypt | 44.2  (35.6-52.7) | 46.4  (38.6-54.2) | 9.4  (5.6-13.3) | 56.7  (47.8-65.6) | 35.1  (28.4-41.9) | 8.1  (5.2-11.1) |
| Iraq | 38.0  (34.2-41.7) | 48.9  (45.0-52.8) | 13.1  (10.9-15.3) | 51.8  (47.3-56.2) | 40.8  (36.6-45.0) | 7.4  (5.4-9.5) |
| Kuwait | 22.1  (15.0-29.1) | 65.9  (60.2-71.6) | 12.0  (8.3-15.7) | 28.0  (23.3-32.7) | 60.6  (56.7-64.4) | 11.4  (9.5-13.3) |
| Lebanon | 32.2  (27.8-36.5) | 58.1  (53.2-63.1) | 9.7  (6.7-12.8) | 38.3  (34.8-41.9) | 54.4  (50.3-58.6) | 7.2  (4.6-9.9) |
| Morocco | 54.3  (49.9-58.6) | 36.4  (32.6-40.1) | 9.4  (6.7-12.1) | 56.1  (50.8-61.5) | 35.0  (31.9-38.1) | 8.8  (6.0-11.7) |
| Oman | 27.6  (25.3-30.0) | 61.2  (58.7-63.7） | 11.1  (9.9-12.4) | 29.3  (23.3-35.3) | 57.7  (54.2-61.2) | 13.0  (9.1-16.8) |
| Pakistan | 79.3  (74.9-83.7) | 18.8  (14.6-23.0) | 1.9  (1.3-2.5) | 78.6  (74.7-82.4) | 20.6  (16.8-24.4) | 0.8  (0.7-0.9) |
| Qatar | 16.4  (12.9-19.9) | 58.9  (54.2-63.7) | 24.7  (19.2-30.1) | 12.1  (9.1-15.1) | 57.6  (52.1-63.0) | 30.3  (25.9-34.7) |
| Sudan | 56.1  (48.9-63.4) | 34.6  (27.8-41.5) | 9.2  (7.0-11.4) | 62.2  (55.0-69.3) | 31.2  (25.0-37.3) | 6.7  (4.8-8.5) |
| Syrian Arab Republic | 55.3  (50.3-60.4) | 38.9  (34.5-43.2) | 5.8  (4.4-7.2) | 59.2  (50.9-67.5) | 36.7  (29.2-44.2) | 4.1  (2.8-5.4) |
| United Arab Emirates | 33.4  (28.8-38.0) | 55.9  (50.7-61.1) | 10.7  (8.1-13.2) | 34.4  (30.8-38.1) | 55.7  (52.8-58.5) | 9.9  (7.8-12.0) |
| Pooled estimates | 40.9  (30.5-51.4) | 47.1  (38.5-55.7) | 10.5  (7.3-13.6) | 45.7  (33.3-58.0) | 44.5  (36.8-52.3) | 9.6  (6.1-13.0) |
| I^2^(%) | 98.5 | 96.9 | 97.1 | 98.8 | 97.5 | 98.2 |
| **Southeast Asia Region** | | | | | | |
| Bangladesh | 43.6  (37.6-49.5) | 44.3  (38.5-50.1) | 12.1  (8.4-15.8) | 51.9  46.6-57.2) | 40.6  (35.7-45.4) | 7.5  (5.3-9.7) |
| Indonesia | 46.9  (43.6-50.1) | 47.5  (44.6-50.4) | 5.6  (4.7-6.6) | 43.8  (41.4-46.2) | 50.9  (48.6-53.2) | 5.3  (4.6-6.0) |
| Thailand | 23.1  (20.0-26.2) | 38.0  (35.3-40.8) | 38.9  (35.4-42.3) | 16.7  (14.6-18.8) | 35.7  (31.8-39.7) | 47.6  (42.7-52.5) |
| Timor-Leste | 34.8  (29.6-40.0) | 53.0  (48.4-57.5) | 12.2  (8.6-15.8) | 30.1  (25.2-34.9) | 58.0  (54.1-61.8) | 12.0  (9.3-14.6) |
| Pooled estimates | 37.0  (24.3-49.7) | 45.6  (38.9-52.3) | 17.2  (2.8-31.6) | 35.6  (18.6-52.5) | 46.4  (37.3-55.5) | 17.8  (7.3-28.2) |
| I^2^(%) | 97.4 | 92.3 | 99.1 | 99.1 | 96.2 | 99.0 |
| **Western Pacific Region** | | | | | | |
| Brunei Darussalam | 34.4  (31.1-37.7) | 58.7  (54.8-62.5) | 6.9  (5.3-8.6) | 33.3  (30.4-36.2) | 60.6  (57.6-63.7) | 6.1  (4.3-7.9) |
| Cambodia | 74.8  (69.5-80.1) | 23.7  (18.9-28.5) | 1.5  (0.2-2.8) | 74.8  (70.9-78.6） | 23.9  (20.6-27.1) | 1.4  (0.4-2.3) |
| Kiribati | 53.4  (48.3-58.5) | 34.0  (30.0-37.9) | 12.6  (9.9-15.4) | 58.1  (53.2-62.9) | 30.6  (26.7-34.5) | 11.3  (8.3-14.3) |
| Lao People's  Democratic Republic | 51.7  (44.4-59.0) | 44.3  (37.8-50.7) | 4.0  (2.3-5.7) | 57.8  (52.6-63.0) | 38.7  (33.6-43.8) | 3.5  (1.9-5.0) |
| Malaysia | 52.2  (49.9-54.4) | 44.9  (42.8-47.1) | 2.9  (2.5-3.3) | 51.1  (49.1-53.0) | 47.0  (45.1-48.8) | 2.0  (1.5-2.4) |
| Mongolia | 44.1  (38.5-49.7) | 39.0  (36.3-41.6) | 16.9  (12.7-21.2) | 45.7  (39.9-51.5) | 36.6  (33.5-39.7) | 17.7  (13.3-22.1) |
| Philippines | 48.5  (42.3-54.7) | 47.3  (42.2-52.3) | 4.2  (2.4-6.1) | 47.7  (41.0-54.3) | 48.4  (42.6-54.2) | 4.0  (2.3-5.6) |
| Samoa | 21.8  (18.3-25.3) | 53.0  (49.7-56.3) | 25.2  (22.2-28.3) | 19.9  (16.1-23.7) | 53.9  (49.1-58.7) | 26.2  (23.6-28.8) |
| Solomon Islands | 35.5  (26.9-44.1) | 48.0  (42.2-53.9) | 16.4  (10.3-22.5) | 32.3  (20.5-44.2) | 44.1  (33.9-54.3) | 23.6  (15.3-31.8) |
| Tonga | 31.2  (27.7-34.6) | 55.4  (51.8-59.1) | 13.4  (10.4-16.4) | 29.4  (25.7-33.0) | 55.4  (51.9-58.8) | 15.3  (12.4-18.1) |
| Vanuatu | 43.3  (30.0-56.6) | 45.1  (31.9-58.3) | 11.5  (7.5-15.6) | 43.6  (33.7-53.4) | 49.2  (40.2-58.2) | 7.2  (4.1-10.3) |
| Vietnam | 70.9  (66.2-75.6) | 24.0  (19.6-28.4) | 5.1  (2.1-8.1) | 69.9  (64.7-75.1) | 24.7  (20.3-29.1) | 5.4  (3.0-7.8) |
| Wallis and Futuna | 34.2  (28.8-39.5) | 43.1  (37.7-48.6) | 22.7  (18.5-26.9) | 35.2  (29.8-40.6) | 37.6  (31.8-43.4) | 27.2  (21.2-33.3) |
| Pooled estimates | 45.9  (37.2-54.5) | 43.1  (37.4-48.8) | 10.7  (7.6-13.8) | 43.7  (35.5-52.0) | 42.3  (35.5-49.1) | 10.9  (7.7-14.1) |
| I^2^(%) | 97.9 | 96.2 | 97.4 | 97.7 | 97.5 | 97.9 |
| **Total** | | | | | | |
| Pooled estimates | 43.9  (40.4-47.3) | 44.6  (42.0-47.3) | 10.7  (8.1-13.4) | 43.5  (39.3-47.7) | 45.4  (42.2-48.5) | 10.0  (8.3-11.7) |
| I^2^(%) | 0.0 | 0.0 | 69.8 | 0.0 | 0.0 | 25.1 |

**Supplementary Table 4.** The weighted prevalence of Fast-Food Consumption Among Adolescents Aged 12–15 Years, by Region, Country and Age.

|  | **12-13 years, %** **(95%CI)** | | | **14-15years, %(95%CI)** | | |
| --- | --- | --- | --- | --- | --- | --- |
|  | **0 days** | **1-3 days** | **4-7 days** | **0 days** | **1-3 days** | **4-7 days** |
| **Africa Region** | | | | | | |
| Algeria | 48.1  (44.5-51.8) | 40.0  (37.4-42.6) | 11.9  (9.4-14.4) | 47.9  (43.0-52.7) | 39.0  (34.9-43.1) | 13.1  (10.6-15.6) |
| Benin | 45.8  (42.1-49.5) | 38.4  (34.3-42.4) | 15.8  (13.6-18.1) | 48.9  (44.1-53.7) | 39.1  (35.3-43.0) | 12.0  (9.2-14.8) |
| Mauritania | 35.9  (25.1-46.6) | 46.6  (38.6-54.5) | 17.6  (11.6-23.5) | 37.4  (31.3-43.5) | 43.7  (39.7-47.7) | 18.9  (15.3-22.5) |
| Mauritius | 48.9  (44.5-53.4) | 47.4  (43.3-51.4) | 3.7  (2.6-4.8) | 43.8  (41.5-46.0) | 50.4  (48.3-52.5) | 5.8  (4.0-7.6) |
| Mozambique | 35.2  (23.8-46.5) | 52.9  (44.4-61.4) | 12.0  (4.1-19.8) | 34.0  (23.3-44.7) | 53.3  (44.4-62.3) | 12.7  8.9-16.5) |
| Namibia | 45.1  (36.9-53.3) | 41.2  (34.3-48.1) | 13.8  (10.1-17.5) | 46.6  (40.8-52.3) | 39.3  (35.5-43.1) | 14.1  (10.5-17.8) |
| Seychelles | 28.8  (24.7-33.0) | 51.9  (48.1-55.7) | 19.3  (15.5-23.0) | 30.1  (26.1-34.2) | 53.5  (50.4-56.5) | 16.4  (12.5-20.3) |
| United Republic of Tanzania | 61.3  (56.2-66.4) | 27.3  (23.6-31.0) | 11.4  (8.4-14.4) | 66.8  (60.4-73.3) | 22.4  (18.5-26.3) | 10.7  (6.6-14.8) |
| Pooled estimates | 44.0  (36.7-51.3) | 42.9  (36.9-49.0) | 13.1  (8.2-17.9) | 44.6  (38.0-51.2) | 42.4  (35.4-49.5) | 12.8  (9.6-16.1) |
| I^2^(%) | 93.6 | 93.4 | 96.0 | 93.5 | 96.7 | 89.2 |
| **Americas Region** | | | | | | |
| Antigua and Barbuda | 40.7  (35.1-46.3) | 48.9  (43.3-54.5) | 10.4  (8.1-12.7) | 44.7  (40.4-49.0) | 44.3  (40.0-48.7) | 11.0  (7.8-14.2) |
| Argentina | 66.1  (62.4-69.9) | 30.1  (26.6-33.5) | 3.8  (2.6-5.0) | 69.6  (67.8-71.4) | 27.3  (25.5-29.0) | 3.1  (2.6-3.7) |
| Barbados | 35.8  (30.4-41.2) | 52.4  (46.6-58.1) | 11.8  (8.2-15.5) | 38.3  (34.3-42.4) | 52.4  (48.9-55.9) | 9.3  (7.3-11.3) |
| Belize | 32.3  (26.8-37.8) | 51.5  (46.9-56.1) | 16.1  (11.8-20.5) | 35.3  (30.2-40.4) | 49.9  (45.0-54.7) | 14.9  (11.7-18.0) |
| Bolivia | 43.0  (39.4-46.5) | 47.3  (43.1-51.6) | 9.7  (7.8-11.5) | 42.8  (40.2-45.3) | 49.2  (46.6-51.7) | 8.1  (6.6-9.6) |
| Chile | 68.7  (64.5-73.0) | 29.3  (25.0-33.5) | 2.0  (1.1-2.9) | 67.0  (62.9-71.1) | 30.6  (27.0-34.2) | 2.5  (0.9-4.0) |
| Bahamas | 29.9  (24.6-35.3) | 55.3  (50.2-60.4) | 14.7  (12.1-17.3) | 25.5  (19.3-31.8) | 56.8  (52.0-61.6) | 17.6  (13.7-21.6) |
| Costa Rica | 49.4  (43.5-55.3) | 43.9  (39.1-48.7) | 6.7  (4.5-8.9) | 43.7  (37.9-49.5) | 49.9  (44.4-55.4) | 6.4  (5.0-7.9) |
| Curaçao | 31.3  (27.3-35.3) | 59.8  (55.5-64.2) | 8.9  (6.5-11.3) | 28.6  (25.7-31.5) | 60.0  (57.2-62.8) | 11.5  (9.4-13.5) |
| El Salvador | 47.0  (41.7-52.3) | 43.7  (38.2-49.3) | 9.3  (6.0-12.6) | 40.5  (36.9-44.2) | 49.6  (46.4-52.8) | 9.9  (7.7-12.0) |
| Guatemala | 42.0  (34.4-49.7) | 51.8  (44.8-58.8) | 6.1  (4.6-7.7) | 43.2  (35.1-51.4) | 49.2  (42.8-55.6) | 7.6  (5.0-10.2) |
| Guyana | 41.9  (38.2-45.6) | 46.0  (41.2-50.8) | 12.1  (8.1-16.0) | 44.9  (41.1-48.6) | 45.3  (41.8-48.8) | 9.8  (8.0-11.6) |
| Honduras | 51.3  (46.8-55.8) | 41.0  (37.5-44.6) | 7.7  (5.9-9.4) | 52.6  (47.4-57.9) | 40.9  (35.5-46.3) | 6.5  (4.8-8.2) |
| Peru | 48.8  (43.3-54.2) | 47.0  (40.8-53.2) | 4.3  (2.7-5.9) | 50.4  (45.7-55.0) | 45.2  (40.6-49.7) | 4.5  (3.4-5.6) |
| Suriname | 35.4  (29.2-41.6) | 54.3  (48.8-59.9) | 10.3  (7.0-13.6) | 39.2  (34.0-44.4) | 53.2  (48.0-58.3) | 7.6  (5.2-10.0) |
| Trinidad and Tobago | 40.0  (35.9-44.2) | 52.9  (49.0-56.8) | 7.1  (5.0-9.1) | 31.6  (26.4-36.8) | 57.3  (53.6-60.9) | 11.1  (8.9-13.4) |
| Uruguay | 55.7  (52.2-59.2) | 40.8  (37.3-44.3) | 3.5  (2.8-4.2) | 55.1  (52.1-58.2) | 40.7  (37.6-43.8) | 4.2  (3.1-5.2) |
| Pooled estimates | 44.7  (39.0-50.4) | 46.7  (42.4-51.1) | 8.2  (6.5-9.9) | 44.4  (37.1-51.6) | 47.1  (41.5-52.7) | 8.3  (6.7-10.0) |
| I^2^(%) | 96.1 | 93.4 | 93.8 | 98.4 | 97.7 | 94.8 |
| **Eastern Mediterranean Region** | | | | | | |
| Afghanistan | 32.4  (24.3-40.4) | 57.8  (50.8-64.9) | 9.8  (6.4-13.2) | 38.8  (31.5-46.1) | 50.0  (43.7-56.3) | 11.2  (8.1-14.3) |
| Egypt | 52.1  (45.3-58.8) | 40.4  (35.6-45.2) | 7.6  (4.2-10.9) | 49.0  (40.7-57.2) | 41.0  (33.1-48.9) | 10.0  (6.9-13.1) |
| Iraq | 44.3  (38.5-50.2) | 45.2  (40.1-50.3) | 10.5  (7.5-13.4) | 44.1  (39.0-49.2) | 45.4  (41.3-49.6) | 10.5  (7.8-13.1) |
| Kuwait | 24.1  (16.6-31.7) | 65.6  (59.9-71.3) | 10.2  (6.0-14.4) | 25.3  (20.3-30.3) | 62.5  (58.3-66.6) | 12.3  (9.3-15.2) |
| Lebanon | 34.7  (30.2-39.2) | 56.9  (52.7-61.2) | 8.4  (5.7-11.1) | 36.0  (32.6-39.4) | 55.6  (51.3-60.0) | 8.4  (4.9-11.8) |
| Morocco | 54.2  (48.8-59.5) | 37.1  (33.9-40.3) | 8.7  (5.7-11.7) | 56.1  (51.6-60.5) | 34.6  (31.0-38.2) | 9.3  (6.9-11.7) |
| Oman | 23.2  (16.9-29.5) | 60.8  (56.6-64.9) | 16.0  (11.1-21.0) | 29.7  (26.4-33.1) | 59.2  (56.3-62.2) | 11.0  (9.0-13.1) |
| Pakistan | 77.1  (71.7-82.4) | 21.8  (16.5-27.2) | 1.1  (0.2-1.9) | 79.6  (76.0-83.2) | 18.8  (15.4-22.1) | 1.6  (1.0-2.2) |
| Qatar | 14.2  (11.4-16.9) | 58.7  (54.4-63.1) | 27.1  (23.9-30.3) | 14.3  (10.9-17.7) | 58.0  (52.7-63.3) | 27.7  (22.8-32.6) |
| Sudan | 64.3  (56.9-71.8) | 27.3  (21.7-32.8) | 8.4  (5.5-11.3) | 57.1  (49.9-64.3) | 35.1  (29.4-40.8) | 7.8  (4.1-11.5) |
| Syrian Arab Republic | 54.8  (47.7-61.9) | 40.4  (33.9-46.9) | 4.8  (3.7-5.9) | 59.4  (54.2-64.6) | 35.6  (30.7-40.4) | 5.1  (3.8-6.4) |
| United Arab Emirates | 31.9  (26.2-37.6) | 58.3  (53.6-63.0) | 9.7  (7.5-12.0) | 35.1  (30.0-40.2) | 54.4  (49.7-59.2) | 10.5  (8.4-12.6) |
| Pooled estimates | 42.2  (30.1-54.4) | 47.5  (39.8-55.3) | 10.1  (6.6-13.6) | 43.7  (31.8-55.5) | 45.9  (37.2-54.5) | 10.2  (7.0-13.4) |
| I^2^(%) | 98.3 | 96.8 | 96.8 | 98.8 | 97.9 | 96.8 |
| **Southeast Asia Region** | | | | | | |
| Bangladesh | 49.9  (43.6-56.3) | 41.4  (36.4-46.3) | 8.7  (5.7-11.7) | 45.2  (39.4-50.9) | 43.6  (37.9-49.2) | 11.2  (7.7-14.8) |
| Indonesia | 45.0  (42.4-47.6) | 49.9  (47.5-52.3) | 5.1  (4.6-5.7) | 45.7  (42.9-48.5) | 48.5  (46.1-50.9) | 5.8  (4.9-6.7) |
| Thailand | 20.2  (17.6-22.8) | 39.6  (35.3-44.0) | 40.1  (35.3-44.9) | 19.7  (17.1-22.3) | 34.6  (32.2-36.9) | 45.8  (42.3-49.2) |
| Timor-Leste | 28.1  (24.1-32.1) | 58.2  (54.1-62.3) | 13.7  (10.2-17.2) | 34.7  (30.0-39.3) | 54.2  (49.9-58.6) | 11.1  (7.9-14.3) |
| Pooled estimates | 35.7  (21.4-49.9) | 47.4  (39.9-54.9) | 16.7  (5.5-27.9) | 36.3  (21.7-50.8) | 45.2  (36.0-54.3) | 18.4  (2.2-34.7) |
| I^2^(%) | 98.6 | 93.6 | 98.7 | 98.5 | 96.8 | 99.4 |
| **Western Pacific Region** | | | | | | |
| Brunei Darussalam | 35.8  (32.3-39.4) | 57.4  (53.4-61.3) | 6.8  (4.8-8.8) | 33.0  (30.3-35.6) | 60.7  (57.6-63.8) | 6.3  (4.8-7.9) |
| Cambodia | 71.1  (63.9-78.3) | 27.2  (19.9-34.5) | 1.7  (0.2-3.2) | 76.0  (72.8-79.1) | 22.7  (19.8-25.6) | 1.3  (0.5-2.2) |
| Kiribati | 49.8  (43.4-56.1) | 34.9  (31.4-38.4) | 15.3  (11.4-19.2) | 58.5  (53.3-63.8) | 31.1  (27.0-35.2) | 10.4  (7.8-13.0) |
| Lao People's  Democratic Republic | 52.6  (43.1-62.1) | 45.5  (36.7-54.4) | 1.8  (0.0-4.7) | 55.5  (49.9-61.1) | 40.4  (35.2-45.7) | 4.1  (2.6-5.5) |
| Malaysia | 51.2  (48.5-53.9) | 46.4  (43.7-49.1) | 2.4  (1.9-2.9) | 51.9  (49.8-53.9) | 45.7  (43.8-47.6) | 2.4  (1.9-2.9) |
| Mongolia | 44.8  (38.8-50.8) | 37.1  (34.3-39.9) | 18.1  (13.5-22.7) | 45.0  (39.2-50.9) | 38.2  (35.1-41.4) | 16.7  (12.5-21.0) |
| Philippines | 44.8  (38.0-51.6) | 50.3  (44.7-55.9) | 4.9  (2.8-7.0) | 49.9  (44.3-55.4) | 46.5  (41.5-51.5) | 3.6  (2.4-4.8) |
| Samoa | 21.7  (16.6-26.8) | 52.1  (48.1-56.0) | 26.2  (21.5-31.0) | 20.8  (17.3-24.4) | 53.7  (49.6-57.8) | 25.5  (22.8-28.2) |
| Solomon Islands | 40.0  (27.2-52.8) | 43.7  (33.8-53.6) | 16.2  (7.5-25.0) | 32.3  (23.1-41.4) | 47.1  (40.3-54.0) | 20.6  (15.8-25.4) |
| Tonga | 30.0  (25.1-35.0) | 54.8  (50.7-58.9) | 15.2  (10.8-19.7) | 30.3  (27.5-33.1) | 55.7  (52.5-58.8) | 14.1  (11.7-16.4) |
| Vanuatu | 43.0  (26.6-59.4) | 46.5  (31.0-61.9) | 10.5  (6.0-15.1) | 44.1  (36.2-52.0) | 47.8  (39.9-55.6) | 8.1  (4.7-11.5) |
| Vietnam | - | - | - | 70.4  (66.4-74.3) | 24.3  (21.1-27.6) | 5.3  (3.1-7.5) |
| Wallis and Futuna | 32.7  (27.3-38.1) | 42.6  (36.8-48.4) | 24.7  (19.9-29.4) | 36.4  (30.8-42.0) | 38.0  (33.6-42.4) | 25.6  (20.3-30.9) |
| Pooled estimates | 43.0  (35.7-50.3) | 45.0  (39.8-50.1) | 11.4  (7.9-14.9) | 46.5  (36.8-56.2) | 42.4  (35.5-49.3) | 10.6  (7.7-13.5) |
| I^2^(%) | 95.1 | 93.2 | 96.6 | 98.8 | 98.0 | 97.9 |
| **Total** | | | | | | |
| Pooled estimates | 43.4  (39.8-46.9) | 45.8  (43.2-48.3) | 10.3  (8.1-12.6) | 44.1  (40.2-48.1) | 44.7  (41.6-47.9) | 10.3  (8.3-12.3) |
| I^2^(%) | 0.0 | 0.0 | 44.3 | 0.0 | 0.0 | 48.8 |

**Supplementary Table 5.** The weighted prevalence of Fast-Food Consumption Among Adolescents Aged 12–15 Years, by Region, Country and Multiple Burden of Malnutrition.

|  | **Stunting** | | | **Thinness** | | | **Overweight** | | | **Obesity** | | |
| --- | --- | --- | --- | --- | --- | --- | --- | --- | --- | --- | --- | --- |
|  | **0 days** | **1-3 days** | **4-7 days** | **0 days** | **1-3 days** | **4-7 days** | **0 days** | **1-3 days** | **4-7 days** | **0 days** | **1-3 days** | **4-7 days** |
| **Africa Region** | | | | | | | | | | | | |
| Algeria | 55.1  (48.9-61.2) | 34.8  (27.4-42.2) | 10.1  (5.7-14.6) | 44.5 (34.8-54.2) | 40.6 (32.3-48.9) | 14.9 (8.2-21.7) | 46.2 (35.1-57.2) | 41.3 (32.8-49.9) | 12.5 (7.3-17.7) | 41.0 (33.1-48.8) | 42.9 (33.0-52.7) | 16.2 (8.3-24.0) |
| Benin | 45.2  (31.7-58.8) | 37.9  (22.3-53.5) | 16.9  (7.6-26.1) | 53.5 (33.7-73.3) | 40.9 (20.1-61.7) | 5.6 (3.1-8.1) | 47.8 (34.2-61.4) | 40.1 (29.5-50.6) | 12.1 (7.2-17.1) | 40.9 (24.6-57.1) | 46.5 (31.8-61.2) | 12.7 (3.0-22.4) |
| Mauritania | 31.2  (28.1-34.3) | 55.5  (47.7-63.3) | 13.3 (2.4-24.2) | 47.0 (36.8-57.2) | 36.2 (16.2-56.2) | 16.8 (0.0-36.3) | 39.5 (27.1-51.8) | 42.5 (30.8-54.1) | 18.1 (9.8-26.4) | 36.6 (18.5-54.6) | 46.5 (28.6-64.3) | 16.9 (0.0-34.7) |
| Mauritius | 45.1  (35.8-54.4) | 51.4  (42.3-60.5) | 3.6 (1.2-5.9) | 48.1 (30.8-65.3) | 43.0 (31.0-55.1) | 8.9 (0.4-17.4) | 53.8 (45.2-62.3) | 42.4 (34.1-50.7) | 3.9 (0.0-8.0) | 56.5 (41.3-71.7) | 41.4 (26.2-56.6) | 2.1 (1.7-2.4) |
| Mozambique | - | - | - | - | - | - | 52.9 (2.8-100.0) | 47.1 (0.0-97.2) | - | - | - | - |
| Namibia | 54.9  (43.8-66.0) | 22.9  (14.8-31.0) | 22.2 (9.8-34.5) | 40.3 (28.9-51.6) | 51.9 (41.0-62.7) | 7.9 (1.8-13.9) | 46.6 (37.3-55.9) | 38.9 (29.1-48.7) | 14.5 (9.0-20.1) | 53.8 (41.6-66.0) | 37.2 (26.8-47.6) | 9.0 (6.5-11.5) |
| Seychelles | 17.9  (7.1-28.6) | 52.2  (39.3-65.0) | 30.0 (20.2-39.7) | 23.3 (13.3-33.2) | 55.4 (44.6-66.1) | 21.4 (11.2-31.6) | 35.2 (27.8-42.6) | 48.9 (42.2-55.6) | 15.9 (9.6-22.3) | 38.1 (30.0-46.3) | 47.2 (36.7-57.8) | 14.6 (6.5-22.8) |
| United Republic of Tanzania | - | - | - | - | - | - | - | - | - | 52.9 (11.0-94.8) | 37.0 (0.0-100.0) | 10.1 (0.0-36.3) |
| Pooled estimates | 41.5 (29.8-53.3) | 42.4 (31.1-53.7) | 15.1 (7.4-22.7) | 41.6 (32.7-50.5) | 45.9 (40.0-51.9) | 11.0 (6.1-15.9) | 44.8 (38.8-50.7) | 43.4 (39.8-47.0) | 12.3 (7.9-16.7) | 43.9 (38.1-49.6) | 43.0 (38.1-48.0) | 10.4 (5.1-15.8) |
| I^2^ (%) | 93.2 | 88.4 | 88.3 | 68.9 | 29.5 | 66.8 | 49.4 | 0.0 | 74.0 | 28.9 | 0.0 | 89.6 |
| **Region of the Americas** | | | | | | | | | | | | |
| Antigua and Barbuda | 48.3 (0.0-100.0) | 51.7（0.0-100.0） | - | 20.7 (15.5-25.9) | 64.3 (55.4-73.3) | 15.0 (11.2-18.8) | 47.6 (34.5-60.8) | 34.1 (18.9-49.4) | 18.2 (0.0-43.9) | 37.6 (27.2-47.9) | 49.9 (27.9-71.9) | 12.5 (0.0-36.2) |
| Argentina | 67.9 (62.2-73.6) | 29.9 (24.3-35.5) | 2.2 (1.4-2.9) | 60.4 (56.4-64.4) | 38.2 (34.2-42.2) | 1.4 (0.6-2.1) | 72.2 (68.2-76.3) | 25.9 (21.8-30.0) | 1.8 (0.7-3.0) | 75.8 (69.8-81.8) | 22.6 (16.5-28.7) | 1.6 (0.3-3.0) |
| Barbados | 40.2 (26.7-53.6) | 49.8 (36.3-63.3) | 10.0 (2.6-17.5) | 33.0 (18.0-48.0) | 51.5 (36.5-66.5) | 15.6 (8.9-22.2) | 50.2 (41.9-58.5) | 46.6 (37.7-55.5) | 3.2 (0.6-5.8) | 40.4 (29.5-51.3) | 52.8 (41.2-64.5) | 6.8 (0.6-12.9) |
| Belize | 46.3 (24.4-68.1) | 40.0 (24.9-55.2) | 13.7 (0.0-27.8) | 27.9 (17.9-38.0) | 59.7 (47.9-71.5) | 12.4 (4.3-20.5) | 38.9 (32.3-45.6) | 50.1 (42.5-57.7) | 11.0 (4.3-17.7) | 34.3 (25.4-43.2) | 50.8 (41.0-60.7) | 14.9 (6.5-23.2) |
| Bolivia | 47.2 (37.9-56.5) | 48.2 (38.9-57.5) | 4.7 (1.6-7.7) | 41.6 (28.9-54.3) | 53.4 (40.5-66.2) | 5.1 (0.6-9.5) | 48.9 (42.1-55.8) | 44.8 (39.3-50.4) | 6.2 (3.5-9.0) | 48.8 (40.4-57.3) | 44.1 (35.5-52.7) | 7.1 (3.5-10.7) |
| Chile | 71.2 (58.9-83.4) | 27.7 (16.2-39.2) | 1.1 (0.0-3.8) | 58.4 (51.6-65.3) | 36.2 (28.1-44.2) | 5.4 (0.0-11.0) | 69.8 (62.5-77.1) | 28.2 (20.9-35.5) | 2.0 (0.2-3.7) | 76.6 (65.7-87.4) | 22.4 (11.4-33.3) | 1.1 (0.8-1.3) |
| Bahamas | 31.3 (15.0-47.6) | 51.5 (34.1-69.0) | 17.2 (5.2-29.1) | 27.5 (14.1-40.8) | 54.1 (40.6-67.7) | 18.4 (5.6-31.2) | 26.9 (16.2-37.6) | 59.7 (46.1-73.3) | 13.4 (5.0-21.7) | 15.0 (6.1-23.9) | 67.1 (53.8-80.5) | 17.8 (7.3-28.3) |
| Costa Rica | 46.7 (34.7-58.7) | 50.0 (38.1-62.0) | 3.3 (0.0-6.7) | 44.3 (27.9-60.8) | 50.0 (33.5-66.5) | 5.6 (2.0-9.2) | 50.5 (41.8-59.2) | 44.5 (36.1-52.9) | 5.0 (2.5-7.6) | 39.2 (32.6-45.8) | 55.4 (48.6-62.2) | 5.4 (0.9-10.0) |
| Curaçao | 35.5 (20.0-51.0) | 50.3 (40.5-60.2) | 14.2 (4.4-23.9) | 26.8 (9.8-43.9) | 67.9 (49.3-86.5) | 5.3 (0.0-10.9) | 33.6 (24.5-42.8) | 58.7 (48.1-69.3) | 7.7 (2.8-12.6) | 32.4 (17.7-47.1) | 47.2 (30.3-64.1) | 20.4 (6.3-34.5) |
| El Salvador | 55.4 (36.7-74.0) | 32.5 (10.8-54.2) | 12.2 (0.9-23.4) | 42.7 (30.7-54.8) | 51.7 (40.1-63.2) | 5.6 (0.0-11.5) | 38.8 (29.8-47.8) | 49.3 (39.6-59.0) | 11.9 (5.9-17.9) | 44.8 (33.1-56.6) | 47.1 (35.4-58.8) | 8.0 (0.3-15.7) |
| Guatemala | 49.7 (34.7-64.8) | 50.2 (35.1-65.2) | 0.1 (0.0-0.3) | 48.1 (44.4-51.8) | 51.0 (47.4-54.5) | 0.9 (0.1-1.8) | 49.5 (42.8-56.3) | 45.7 (38.0-53.5) | 4.7 (0.1-9.4) | 43.1 (33.5-52.7) | 52.3 (44.9-59.7) | 4.6 (2.4-6.9) |
| Guyana | 38.0 (28.7-47.2) | 44.6 (29.5-59.7) | 17.4 (3.9-31.0) | 45.0 (32.0-58.0) | 43.8 (32.8-54.8) | 11.3 (4.8-17.8) | 46.3 (35.1-57.6) | 45.8 (35.0-56.6) | 7.9 (5.2-10.6) | 52.7 (44.4-61.0) | 41.8 (34.0-49.7) | 5.5 (0.0-11.8) |
| Honduras | 53.1 (39.6-66.5) | 46.9 (33.5-60.4) | - | 58.9(44.0-73.8) | 30.7 (15.5-45.9) | 10.4 (2.1-18.7) | 54.2 (44.3-64.2) | 36.5 (27.0-46.0) | 9.3 (4.5-14.1) | 49.9 (38.7-61.2) | 44.6 (33.7-55.5) | 5.5 (0.0-11.6) |
| Peru | 53.3 (43.7-62.8) | 42.1 (32.1-52.1) | 4.7 (1.0-8.4) | 42.2 (36.6-47.7) | 54.8 (48.0-61.6) | 3.0 (0.0-6.9) | 48.7 (41.9-55.4) | 44.7 (37.0-52.4) | 6.7 (3.1-10.3) | 49.5 (41.5-57.5) | 46.3 (38.4-54.2) | 4.2 (0.5-7.9) |
| Suriname | 43.3 (35.8-50.8) | 40.2 (26.6-53.8) | 16.5 (5.7-27.3) | 38.1 (16.3-59.9) | 37.6(16.0-59.3) | 24.3 (5.0-43.5) | 36.7 (24.1-49.2) | 51.2 (39.0-63.4) | 12.1 (7.3-16.9) | 40.0 (20.4-59.6) | 56.1 (41.4-70.8) | 3.9 (0.0-9.8) |
| Trinidad and Tobago | 31.8 (19.1-44.4) | 57.5 (46.1-68.9) | 10.7 (3.4-18.1) | 31.3 (20.5-42.1) | 59.6 (48.6-70.7) | 9.1 (0.7-17.4) | 46.5 (38.3-54.7) | 45.4 (36.3-54.5) | 8.1 (2.0-14.2) | 39.3 (21.2-57.4) | 58.5 (38.8-78.2) | 2.2 (0.0-6.0) |
| Uruguay | 54.5 (43.4-65.5) | 43.6 (32.5-54.7) | 1.9 (0.0-4.8) | 54.1 (44.2-64.0) | 43.2 (33.1-53.4) | 2.6 (0.0-5.6) | 56.8 (48.9-64.6) | 40.9 (33.4-48.5) | 2.3 (0.4-4.2) | 66.3 (56.3-76.3) | 29.3 (19.5-39.2) | 4.4 (1.4-7.4) |
| Pooled estimates | 48.2 (41.8-54.6) | 44.0 (39.1-48.9) | 4.3 (2.6-5.9) | 41.6 (34.5-48.6) | 49.7 (44.9-54.5) | 6.7 (4.7-8.7) | 48.3 (41.7-54.9) | 43.9 (38.8-48.9) | 6.2 (4.5-7.9) | 46.5 (38.1-54.8) | 45.7 (39.4-52.1) | 4.9 (3.3-6.5) |
| I^2^ (%) | 79.7 | 62.2 | 86.3 | 91.9 | 77.9 | 85.8 | 91.6 | 84.3 | 77.9 | 92.2 | 85.2 | 77.2 |
| **Eastern Mediterranean Region** | | | | | | | | | | | | |
| Afghanistan | 51.9 (4.5-99.3) | 41.2 (1.9-80.5) | 6.8 (0.0-15.0) | 37.3 (27.5-47.1) | 54.3 (42.8-65.7) | 8.4 (1.8-15.0) | 36.1 (30.1-42.2) | 54.2 (48.1-60.2) | 9.7 (3.6-15.8) | 44.6 (27.5-61.8) | 46.8 (38.6-55.1) | 8.5 (0.0-20.8) |
| Egypt | 52.6 (39.5-65.7) | 38.6 (27.1-50.0) | 8.8 (4.4-13.2) | 57.0 (41.9-72.2) | 34.2 (18.6-49.9) | 8.7 (4.0-13.5) | 53.3 (42.3-64.3) | 38.9 (30.0-47.7) | 7.8 (3.1-12.6) | 54.2 (41.0-67.4) | 28.9 (19.0-38.7) | 16.9 (9.7-24.2) |
| Iraq | 47.6 (38.2-57.0) | 36.5 (29.5-43.5) | 15.9 (8.7-23.1) | 34.6 (25.5-43.8) | 56.1 (43.0-69.1) | 9.3 (2.9-15.7) | 45.6 (37.5-53.7) | 42.2 (33.8-50.6) | 12.2 (7.3-17.0) | 46.5 (34.6-58.4) | 47.4 (36.9-58.0) | 6.0 (0.7-11.3) |
| Kuwait | 21.1 (8.9-33.2) | 64.1 (52.4-75.7) | 14.9 (7.9-21.9) | 34.2 (25.8-42.7) | 54.0 (44.1-63.9) | 11.7 (6.0-17.5) | 20.7 (10.9-30.4) | 70.6 (63.2-78.1) | 8.7 (4.1-13.3) | 26.4 (16.6-36.2) | 65.2 (55.5-74.8) | 8.4 (2.6-14.3) |
| Lebanon | 33.2 (25.3-41.2) | 54.9 (44.8-65.0) | 11.9 (3.8-20.0) | 31.8 (17.4-46.2) | 65.2 (49.8-80.5) | 3.0 (0.0-7.0) | 43.8 (35.0-52.5) | 48.8 (41.2-56.4) | 7.4 (3.8-11.0) | 46.2 (16.0-76.4) | 50.6 (20.7-80.4) | 3.2 (0.0-7.2) |
| Morocco | 62.4 (51.9-72.9) | 31.0 (19.9-42.2) | 6.6 (1.5-11.6) | 56.4 (48.8-64.0) | 37.8 (31.2-44.5) | 5.8 (1.9-9.6) | 48.8 (44.0-53.6) | 41.9 (36.2-47.6) | 9.3 (5.5-13.2) | 65.3 (55.1-75.5) | 27.3 (17.9-36.6) | 7.4 (1.5-13.4) |
| Oman | 27.1 (19.8-34.4) | 63.2 (55.8-70.7) | 9.7 (2.2-17.2) | 24.4 (19.2-29.5) | 65.9 (59.5-72.2) | 9.8 (4.1-15.4) | 25.8 (18.0-33.6) | 60.6 (52.5-68.7) | 13.6 (7.7-19.6) | 30.0 (21.8-38.2) | 63.3 (54.5-72.1) | 6.7 (2.4-11.0) |
| Pakistan | 84.8 (78.4-91.1) | 14.6 (8.6-20.7) | 0.6 (0.0-1.9) | 82.8 (77.6-88.0) | 15.9 (10.7-21.2) | 1.2 (0.0-3.0) | 78.4 (72.3-84.4) | 20.3 (14.6-25.9) | 1.4 (0.1-2.6) | 70.0 (63.4-76.7) | 27.2 (21.1-33.2) | 2.8 (1.0-4.6) |
| Qatar | 21.0 (16.2-25.7) | 59.1 (45.5-72.7) | 19.9 (9.3-30.5) | 3.5 (0.0-12.1) | 67.1 (62.7-71.4) | 29.5 (19.1-39.9) | 8.3 (1.7-14.9) | 71.2 (60.3-82.1) | 20.6 (11.0-30.1) | 29.2 (17.2-41.2) | 51.3 (35.4-67.2) | 19.5 (11.1-28.0) |
| Sudan | 58.9 (52.3-65.5) | 30.9 (25.2-36.7) | 10.2 (8.9-11.5) | 54.9 (34.0-75.7) | 31.1 (12.8-49.3) | 14.1 (4.4-23.7) | 52.2 (40.0-64.4) | 38.1 (24.1-52.0) | 9.7 (5.3-14.2) | 51.1 (28.0-74.3) | 42.3 (27.6-57.0) | 6.5 (0.0-15.4) |
| Syrian Arab Republic | 61.7 (52.0-71.5) | 36.9 (27.6-46.2) | 1.4 (0.0-3.8) | 53.7 (41.3-66.1) | 40.0 (28.2-51.8) | 6.3 (1.9-10.7) | 53.0 (45.0-60.9) | 42.1 (34.1-50.0) | 5.0 (2.1-7.9) | 53.4 (41.3-65.5) | 38.2 (28.8-47.6) | 8.4 (2.2-14.5) |
| United Arab Emirates | 38.1 (24.5-51.6) | 52.0 (39.6-64.4) | 10.0 (4.5-15.4) | 29.5 (19.2-39.8) | 59.0 (45.5-72.5) | 11.5 (5.5-17.5) | 34.1 (23.2-45.0) | 57.8 (48.2-67.5) | 8.1 (3.0-13.2) | 37.6 (28.8-46.5) | 47.2 (37.3-57.0) | 15.2(9.9-20.6) |
| Pooled estimates | 46.5 (32.6-60.4) | 43.4 (33.3-53.4) | 9.0 (5.4-12.6) | 41.5 (25.6-57.4) | 48.5 (36.0-60.9) | 8.8 (5.5-12.1) | 41.7  (30.2-53.2） | 48.8 (39.9-57.7) | 8.8 (5.9-11.7) | 46.1 (35.9-56.3) | 44.2 (35.9-52.6) | 8.5 (5.5-11.5) |
| I^2^ (%) | 96.7 | 92.8 | 94.2 | 97.7 | 96.0 | 83.7 | 96.4 | 93.7 | 85.6 | 89.7 | 87.7 | 76.0 |
| **Southeast Asia Region** | | | | | | | | | | | | |
| Bangladesh | 43.1 (32.8-53.5) | 40.5 (36.6-44.4) | 16.4 (5.5-27.2) | 54.7 (39.9-69.5) | 33.9 (22.5-45.2) | 11.4 (4.9-17.9) | 54.0 (39.3-68.6) | 37.2 (25.5-48.9) | 8.8 (3.6-14.0) | 57.0 (45.0-69.0) | 34.1 (23.9-44.3) | 8.9 (6.1-11.6) |
| Indonesia | 46.2 (36.6-55.7) | 48.3 (40.8-55.8) | 5.5 (2.4-8.6) | 49.5 (42.4-56.6) | 46.5 (40.1-52.9) | 4.0 (1.5-6.5) | 43.5 (39.4-47.6) | 52.6 (48.5-56.8) | 3.9 (2.3-5.4) | 39.9 (33.6-46.1) | 55.5 (49.5-61.6) | 4.6 (2.3-6.9) |
| Thailand | 31.2 (19.4-43.0) | 45.3 (34.6-55.9) | 23.5 (15.7-31.3) | 22.6 (14.7-30.5) | 37.7 (29.0-46.4) | 39.7 (30.0-49.4) | 23.0 (18.2-27.8) | 37.5 (29.8-45.1) | 39.5 (33.0-46.0) | 16.9 (13.1-20.7) | 35.0 (28.5-41.5) | 48.1 (42.6-53.5) |
| Timor-Leste | 31.4 (16.0-46.8) | 56.5 (23.4-89.6) | 12.1 (0.0-34.1) | 31.7 (12.9-50.6) | 59.4 (43.6-75.3) | 8.8 (2.9-14.8) | 26.6 (21.4-31.8) | 62.3 (54.5-70.2) | 11.0 (6.6-15.4) | 35.6 (17.8-53.4) | 45.9 (29.8-61.9) | 18.5 (4.2-32.8) |
| Pooled estimates | 39.1 (31.5-46.8) | 43.8 (38.9-48.7) | 14.2 (3.5-24.8) | 39.6 (22.8-56.4) | 43.3 (34.7-51.9) | 15.2 (3.8-26.6) | 35.6 (23.4-47.9) | 47.9 (37.2-58.5) | 15.5 (3.2-27.8) | 36.9 (18.5-55.3) | 42.6 (30.3-55.0) | 19.9 (3.7-36.1) |
| I^2^ (%) | 43.4 | 29.5 | 85.0 | 90.0 | 67.2 | 94.2 | 94.6 | 88.3 | 97.4 | 95.6 | 88.0 | 98.6 |
| **Western Pacific Region** | | | | | | | | | | | | |
| Brunei Darussalam | 34.4 (24.4-44.4) | 59.1 (46.9-71.4) | 6.4 (2.5-10.4) | 32.5 (22.7-42.2) | 65.2 (55.4-75.1) | 2.3 (0.0-5.2) | 34.6 (27.0-42.1) | 62.8 (55.3-70.3) | 2.6 (0.2-5.1) | 41.1 (31.7-50.5) | 50.9 (41.6-60.3) | 8.0 (1.6-14.4) |
| Cambodia | 78.5 (68.1-88.8) | 19.3 (8.6-30.1) | 2.2 (0.0-5.7) | 78.1 (72.9-83.2) | 20.3 (15.7-24.8) | 1.7 (0.0-5.4) | 81.4 (74.1-88.7) | 18.4 (11.0-25.7) | 0.3 (0.0-0.8) | 69.0 (63.2-74.7) | 30.4 (24.6-36.1) | 0.7 (0.5-0.9) |
| Kiribati | 50.6 (32.1-69.0) | 32.7 (17.5-47.9) | 16.7 (4.1-29.4) | 61.4(50.8-72.0) | 30.7 (18.9-42.6) | 7.9 (2.0-13.8) | 58.9 (48.6-69.2) | 30.3 (21.4-39.2) | 10.8 (6.3-15.3) | 48.8 (37.6-59.9) | 35.1 (25.4-44.9) | 16.1 (5.9-26.3) |
| Lao People's  Democratic Republic | 53.6 (48.4-58.7) | 41.3 (38.0-44.7) | 5.1 (2.8-7.3) | 53.3 (41.4-65.2) | 45.2 (33.5-57.0) | 1.5 (0.0-4.8) | 54.1 (45.8-62.3) | 41.5 (31.0-52.0) | 4.4 (0.0-9.1) | 53.8 (39.8-67.8) | 44.9 (30.8-59.0) | 1.3 (0.0-4.3) |
| Malaysia | 52.3 (47.7-57.0) | 45.1 (40.3-49.9) | 2.6 (1.0-4.1) | 49.8 (45.4-54.2) | 47.0 (42.3-51.8) | 3.2 (1.7-4.6) | 57.7 (54.7-60.6) | 40.7 (37.8-43.6) | 1.6 (0.8-2.4) | 54.7 (50.3-59.2) | 44.0 (39.6-48.4) | 1.3 (0.6-1.9) |
| Mongolia | 63.2 (53.8-72.7) | 22.0 (14.8-29.1) | 14.8 (7.7-21.9) | 39.8 (29.3-50.3) | 36.4 (30.7-42.1) | 23.8 (14.4-33.3) | 39.2 (33.3-45.2) | 39.7 (33.6-45.8) | 21.0 (15.2-26.9) | 36.0 (28.6-43.4) | 43.9 (35.2-52.6) | 20.1 (11.7-28.5) |
| Philippines | 54.7 (48.8-60.6) | 43.3 (37.5-49.2) | 2.0 (0.2-3.7) | 44.3 (35.0-53.6) | 54.2 (45.4-63.0) | 1.6 (0.3-2.9) | 43.0 (34.9-51.1) | 51.0 (44.5-57.5) | 6.0 (2.5-9.5) | 40.4 (31.4-49.3) | 54.7 (46.2-63.2) | 4.9 (1.2-8.7) |
| Samoa | 21.1 (3.9-38.3) | 43.2 (22.5-63.8) | 35.8 (22.2-49.4) | 28.8 (16.5-41.0) | 47.2 (34.7-59.7) | 24.0 (12.4-35.6) | 19.1 (13.8-24.4) | 62.4 (56.9-68.0) | 18.5 (13.4-23.5) | 16.4 (8.6-24.2) | 60.3 (50.4-70.3) | 23.3 (14.3-32.2) |
| Solomon Islands | 28.2 (0.0-57.4) | 57.5 (57.2-57.7) | 14.3 (0.0-43.1) | 51.0 (1.0-100.0) | 37.0 (0.0-81.3) | 12.0 (5.7-18.3) | 33.4 (12.1-54.8) | 42.5 (28.4-56.6) | 24.1 (9.6-38.5) | 42.6 (18.5-66.7) | 45.7 (26.3-65.2) | 11.7 (5.1-18.3) |
| Tonga | 35.3 (28.1-42.5) | 51.2 (39.4-63.0) | 13.5 (4.2-22.8) | 27.2 (17.3-37.1) | 59.2 (50.1-68.4) | 13.5 (8.2-18.9) | 32.5 (25.0-40.0) | 55.7 (47.9-63.4) | 11.8 (7.3-16.4) | 30.8 (21.2-40.4) | 53.3 (42.5-64.1) | 15.9 (9.9-21.9) |
| Vanuatu | 52.6 (0.0-100.0) | 44.4 (0.0-100.0) | 3.1 (0.3-5.9) | 58.8 (51.8-65.7) | 38.4 (21.5-55.3) | 2.8 (0.0-16.5) | 37.6 (23.8-51.5) | 45.7 (40.3-51.1) | 16.6 (3.7-29.6) | 38.3 (19.7-56.8) | 45.3 (29.6-61.0) | 16.5 (10.0-22.9) |
| Vietnam | 72.3 (58.2-86.4) | 24.6 (10.8-38.5) | 3.0 (0.0-6.0) | 67.1 (53.9-80.3) | 28.1 (18.5-37.7) | 4.8 (0.0-10.9) | 72.3 (64.7-79.9) | 22.2 (16.0-28.4) | 5.5 (2.1-8.9) | 71.3 (59.3-83.4) | 26.3 (14.4-38.3) | 2.3 (1.9-2.7) |
| Wallis and Futuna | 40.4 (12.1-68.8) | 44.0 (14.2-73.8) | 15.5 (0.0-35.1) | 44.2 (22.6-65.7) | 22.1 (1.8-42.5) | 33.7 (9.6-57.8) | 35.5 (24.8-46.3) | 37.2 (25.6-48.8) | 27.3 (14.3-40.3) | 43.4 (25.6-61.3) | 38.8 (18.5-59.1) | 17.7 (6.6-28.8) |
| Pooled estimates | 50.5 (43.0-58.0) | 40.4 (32.1-48.7) | 5.8 (3.6-8.0) | 49.1 (39.4-58.7) | 41.4 (32.7-50.0) | 6.2 (3.8-8.6) | 46.4 (36.1-56.6) | 42.4 (35.2-49.6) | 8.5 (6.1-11.0) | 45.2 (35.7-54.7) | 44.2 (38.5-49.9) | 5.1 (3.6-6.5) |
| I^2^ (%) | 45.6 | 43.3 | 7.6 | 43.0 | 46.7 | 7.7 | 44.9 | 44.1 | 8.6 | 44.7 | 44.0 | 6.6 |
| **Total** | | | | | | | | | | | | |
| Pooled estimates | 45.6 (41.1-50.2) | 43.3 (40.4-46.3) | 7.6 (4.6-10.5) | 43.0 (38.6-47.5) | 46.7 (43.6-49.8) | 7.7 (5.9-9.5) | 44.9 (41.3-48.6) | 44.1 (41.6-46.6) | 8.6 (6.4-10.9) | 44.7 (40.8-48.5) | 44.0 (41.1-46.9) | 6.6 (4.5-8.7) |
| I^2^ (%) | 27.5 | 0.0 | 71.9 | 0.0 | 0.0 | 33.1 | 0.0 | 0.0 | 58.7 | 0.0 | 0.0 | 63.2 |
